# Markers associated with heading and aftermath heading in perennial ryegrass full-sib families

**DOI:** 10.1186/s12870-016-0844-y

**Published:** 2016-07-16

**Authors:** Sai Krishna Arojju, Susanne Barth, Dan Milbourne, Patrick Conaghan, Janaki Velmurugan, Trevor R. Hodkinson, Stephen L. Byrne

**Affiliations:** Teagasc, Crop Science Department, Oak Park, Carlow Ireland; Teagasc, Grassland Science Research Department, Animal and Grassland Research and Innovation Centre, Oak Park, Carlow Ireland; Department of Botany, Trinity College Dublin, Dublin 2, Dublin Ireland

**Keywords:** Aftermath heading, Flowering, Genome wide association, Heading, *Lolium perenne*, Perennial ryegrass, Single marker analysis

## Abstract

**Background:**

Heading and aftermath heading are important traits in perennial ryegrass because they impact forage quality. So far, genome-wide association analyses in this major forage species have only identified a small number of genetic variants associated with heading date that overall explained little of the variation. Some possible reasons include rare alleles with large phenotypic affects, allelic heterogeneity, or insufficient marker density. We established a genome-wide association panel with multiple genotypes from multiple full-sib families. This ensured alleles were present at the frequency needed to have sufficient statistical power to identify associations. We genotyped the panel via partial genome sequencing and performed genome-wide association analyses with multi-year phenotype data collected for heading date, and aftermath heading.

**Results:**

Genome wide association using a mixed linear model failed to identify any variants significantly associated with heading date or aftermath heading. Our failure to identify associations for these traits is likely due to the extremely low linkage disequilibrium we observed in this population. However, using single marker analysis within each full-sib family we could identify markers and genomic regions associated with heading and aftermath heading. Using the ryegrass genome we identified putative orthologs of key heading genes, some of which were located in regions of marker-trait associations.

**Conclusion:**

Given the very low levels of LD, genome wide association studies in perennial ryegrass populations are going to require very high SNP densities. Single marker analysis within full-sibs enabled us to identify significant marker-trait associations. One of these markers anchored proximal to a putative ortholog of TFL1, homologues of which have been shown to play a key role in continuous heading of some members of the rose family, Rosaceae.

**Electronic supplementary material:**

The online version of this article (doi:10.1186/s12870-016-0844-y) contains supplementary material, which is available to authorized users.

## Background

Perennial ryegrass (*Lolium perenne* L.) is an important forage species grown in temperate regions of the world where it underpins the dairy and livestock sectors. This is due to a high palatability and digestibility [1]. It also displays relatively rapid establishment and has long growing seasons with relatively high yields in suitable environments [2]. With 38 % of global land area available for agriculture, 70 % is assigned as pastoral agricultural land [3]. In Europe alone 76 million hectares is used as permanent pasture [4] and in Ireland 80 % agricultural land (3.4 million hectares) is used for pasture, hay and silage where perennial ryegrass is the preferred species [5].

Heading date is a trait that can have a large effect to the use of perennial ryegrass as a forage [6]. Heading date is associated with a reduction in forage quality [7–9]. The stem and inflorescence formation significantly reduces tiller formation and affects the persistency, digestibility and nutritional value [10]. Perennial ryegrass belongs to the same sub-family (Pooideae) as several other important grain cereals such as barley, oats, rye and wheat [11, 12]. Heading in situations outside of seed production is unwanted as it negatively impacts forage quality by increasing the stem to leaf ratio. Extending the vegetative period would greatly enhance its utility as a forage [13, 14]. Aftermath heading is mainly associated with early heading genotypes, and these tend to show lower persistency and perenniality. There has been limited work done on the genetic control of aftermath heading, and only a single quantitative trait locus (QTL) has been mapped onto linkage group (LG) 6 in an experimental mapping population [9].

In perennial ryegrass, heading is mainly controlled by three main pathways, namely the vernalization pathway, the photoperiod pathway and the circadian clock. To date many QTL mapping studies have been carried out in perennial ryegrass and major loci involved in the floral transition have been identified [9, 15–24]. QTL for heading date have been detected on all seven LGs of perennial ryegrass, with analogous regions on LG4 and LG7 being linked with large affect QTL across multiple populations [17]. Although genes underlying some of these QTL have been proposed [16, 17] none have been cloned to date.

In addition to within family based QTL analysis, we can also map QTL in populations using genome wide association analysis (GWAS). This offers the benefit of being able to take advantage of historical recombination to more precisely map the QTL region. In the case of a very rapid decay of linkage disequilibrium (LD), the causative quantitative trait nucleotide (QTN) may be elucidated. However, this does necessitate the need for a high marker density. A recent GWAS study of heading date in perennial ryegrass identified markers affecting heading date across 1,000 F2 families [25]. However, the variation explained by the combined marker set was extremely small. LD only extended to very short distances in the study population, and despite using in excess of 0.9 million SNPs the marker density may be insufficient. Alternatively, rare variants affecting the trait may have resulted in low statistical power to identify associations.

Here, we have developed an association mapping population of 360 individuals coming from six full-sib families with contrasting primary heading dates. Multiple individuals from each full-sib family were selected to ensure any allele will be present at a frequency suitable for association analysis. However, the low levels of LD across families restricted GWAS at our marker density, and so we performed single marker analysis within each full-sib family separately. Anchoring markers to the perennial ryegrass GenomeZipper [26, 27] allowed us to identify regions containing clusters of associated markers, some of which co-located with genes having a known involvement in controlling heading and aftermath heading.

## Results and discussion

### Phenotypic variation for heading date and aftermath heading

**Fig. 1:**
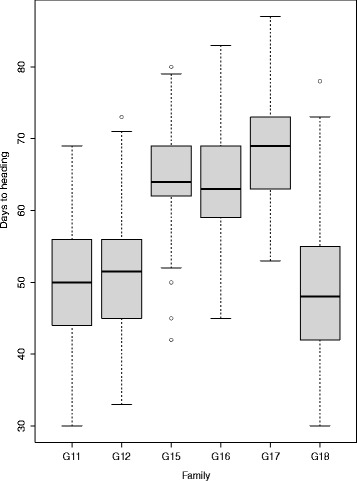
Phenotypic distribution of heading date in six full-sib families. Boxplots representing heading date in full-sib families with y-axis showing days to heading and families on x-axis

The 360 genotypes were planted in two replicates at Oak Park, Carlow, Ireland, and were scored for days to heading in both 2014 and 2015. Spaced plants were scored for the number of days from April 1st until three spikes had emerged on a single plant. In all families, days to heading follows a normal statistical distribution. Plants are generally assigned to one of three groups for heading, these are early (head in first half of May), intermediate (head in second half of May), and late (head in first half of June). The full-sib families G15, G16, and G17 were all developed from late heading parents (Table 1) and this is evident in the phenotypic distributions for these families (Fig. 1). Only G11 had an early heading parent, and G12 and G18 involved intermediate heading parents. Scores for heading date were strongly correlated between 2014 and 2015, with a Pearson’s product-moment correlation of 0.82 with a 95 % confidence interval of (0.79, 0.84). Variance components were calculated using lme4 [28] genotype as a random effect and year as fixed effect. From this, we calculated heritability on a line mean basis to be 0.88.

**Table 1:** Full-sib family structure

	Parent1	Parent2	Crosses
G11	Pastour	Genesis	Late X Early
G12	Solomon	Tyrella	Inter X Late
G15	Profit X Hercules	Jumbo X Tyrone	Late X Late X Late X Late
G16	AberAvon	Twystar	Late X Late
G17	Tyrconnell	Majestic	Late X Late
G18	AberSilo	Shandon	Inter X Inter

Aftermath heading was scored only in September 2015 using a visual assessment on a scale of 1 (no aftermath-heading) to 9 (extensive aftermath heading). The Pearson’s product-moment correlation between replicates was 0.68 and a 95 % confidence interval of (0.61, 0.73). The difference in aftermath heading scores between replicates was not significant(*F*_(1,685)_= 3.385, *MSE* =21.522, *P* = 0.07) at *α*= 0.05. The population mean, sd, and median scores for aftermath heading were 2.7, 2.7 and 1, respectively. We only have a single years data for aftermath heading, however, a recent study of 1453 *F*_2_ families of perennial ryegrass determined heritibalities for aftermath heading that were in line with those determined for heading date [29]. The distribution of scores within each full-sib family, we see that one family (G18) has more variation and a higher propensity for aftermath heading. Taking this family in isolation we looked at the association between heading date and aftermath heading. Using aftermath heading as a response variable in linear regression, we can see that earlier heading individuals tend to have higher aftermath heading (Additional file 1).

### Genome wide association analysis

**Fig. 2:**
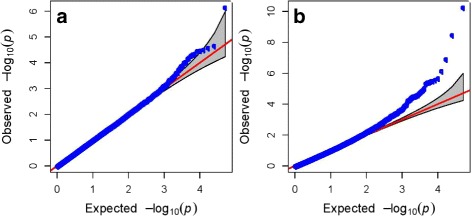
QQ-plots for **a** heading and **b** aftermath heading

We used a genotyping by sequencing approach to characterize variation in the association panel. Data were aligned to the reference genome [30] and variants were identified across the entire 360 genotypes (Table 2). Only variants present in at least 70 % of samples and at a minor allele frequency of 5 % were retained. This left 51,846 SNPs for association analysis with the traits heading date and aftermath heading. We corrected for population structure using principal component analysis and the kinship matrix (Fig. 2). The purpose of including 60 genotypes from each of six full-sib families was to inflate the allele frequencies to ensure we had adequate statistical power for association studies. It is possible that many traits in perennial ryegrass may be controlled by rare alleles with large effects, but in order to detect associations an allele must be present in high enough frequency. Within our association panel the rarest allele would, in theory, be present in 30 individuals (each full-sib family is the result of a single pair cross followed by seed multiplication in isolation plots).

**Table 2:** Markers used at different stages of pipeline

Family	Genotyped	Filtered	Chi-square	Tagged to
	markers	markers	threshold	zipper
All	51,846	-	-	-
G11	27,934	15,315	7070	2174
G12	59,524	28,606	15,564	4228
G15	77,499	32,805	19,425	4424
G16	62,948	29,263	15,563	3421
G17	63,516	27,007	14,315	3523
G18	17,701	6021	3075	1225

**Fig. 3:**
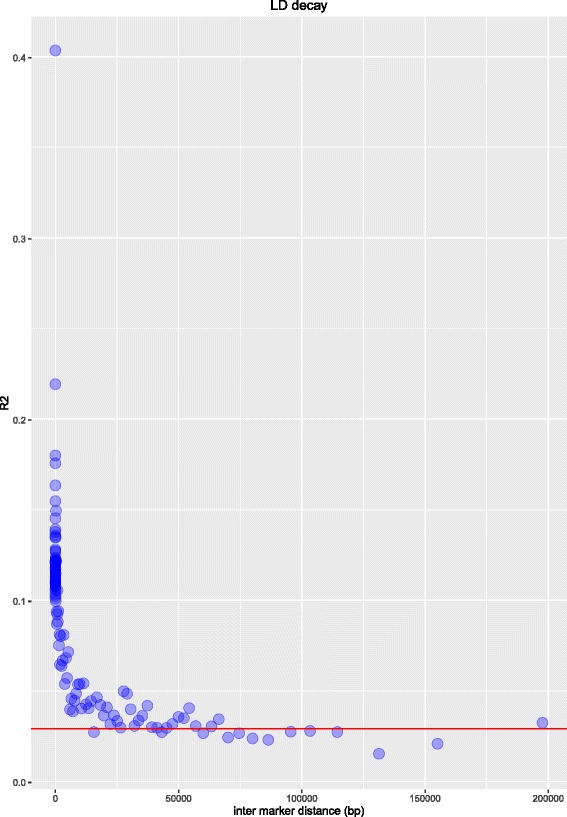
Extent of linkage disequilibrium (LD) measured as the squared correlation of allele counts (y-axis), based on the maximum likelihood solution to the cubic equation. The x-axis shows inter marker distance in bp. LD estimates were sorted according to inter-marker distance, and divided into bins of 1000 estimates. Each point on the plot represents the mean *R* ^2^ and mean inter-marker distance of 1000 measurements

We did not find any markers significantly associated with heading date or aftermath heading after correcting for multiple testing (FDR < 0.05). Heading date is a highly heritable trait [29], and one that can be phenotyped very precisely. It was therefore initially surprising that we did not identify any significant associations. Genome wide association studies can fail for many reasons, including a lack of statistical power due to many rare alleles with large effects or allelic heterogeneity. However, to avoid this problem we have used multiple genotypes (60) from each of six full sib families. Another possible explanation is that heading date (and aftermath heading) is highly correlated with population structure, meaning any correction for population structure will result in false negatives. Designing populations to remove any population structure is complicated for a trait like heading date as synchronization of heading is required for cross pollination. Another possible explanation is that the marker density is insufficient to ensure we always have a marker in LD with a QTL. The six *F*_2_ families were developed from pair-crosses of 12 genotypes taken from a recurrent selection program. When we evaluated the extent of LD in the population, we observed that on average across the genome it decayed very rapidly (Fig. 3). Based on this, our marker density is not sufficient, even considering that our genotyping approach is focused on the non-repetitive and gene-rich fractions of the genome. In this case it is likely that full re-sequencing of the gene space and regions up and downstream is required to capture alleles associated with a trait. A recent GWAS study in perennial ryegrass using almost 20 times the number of markers (∼ 1 million SNPs), did identify significant SNPs for heading. Some SNPs were in close proximity with key heading genes like *CONSTANS* (*CO*) and *PHYTOCHROME C* (*PHYC*), but the sum of the variances explained by all significant markers was only 20.3 % [25].

We have now established that, in general, marker numbers in the region of 50,000 are going to be unsuitable for GWAS in perennial ryegrass. We believe that our inability to find any significant associations with heading date and aftermath heading was due to low marker density and the extremely low LD in the population. Our population is made up of six full-sib families, and within each family a much higher LD is expected. An alternative approach would be to perform single marker analysis within each full-sib family. There are only 60 genotypes per full-sib family, however, using this approach there is sufficient SNP density to perform a simple marker-trait association analysis within each family separately. This would not enable us to locate the regions directly affecting a phenotype, but would allow us to identify markers linked to QTL.

### Single marker analysis in full-sib families

**Fig. 4:**
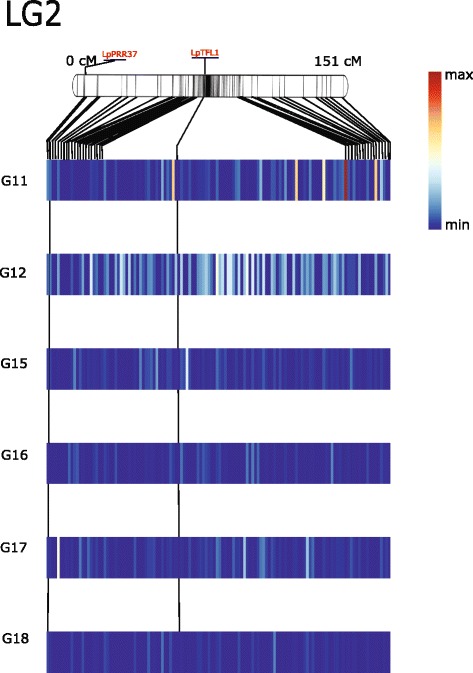
Heatmap illustrates regions associated with heading over six full-sib families on perennial ryegrass LG2. A Kruskal-Wallis test was performed on each marker to identify significant regions for heading. Using the perennial ryegrass genome zipper [26, 30] we identified a putative gene order for markers on LG2. These data were used to construct the heatmap for each family. A perennial ryegrass transcriptome-based genetic linkage map upon which GenomeZipper was based was used as reference to construct LG2 [26, 27] and placed above the heatmap. Each bar in the heatmap represents region between two genetic markers from the linkage map. The median Kruskal-Wallis test statistic was calculated for markers binned between markers on the genetic linkage map and used to construct the heatmap. Putative orthologs of LpPRR37 and LpTFL1, were identified in the phylogenetic analysis and placed onto LG2 using genetic positions from genome zipper. The genetic positions of these orthologs were extrapolated over the heatmap. Color of the heatmap illustrates the test-statistic of the Kruskal-wallis analysis from 0 to 23

**Fig. 5:**
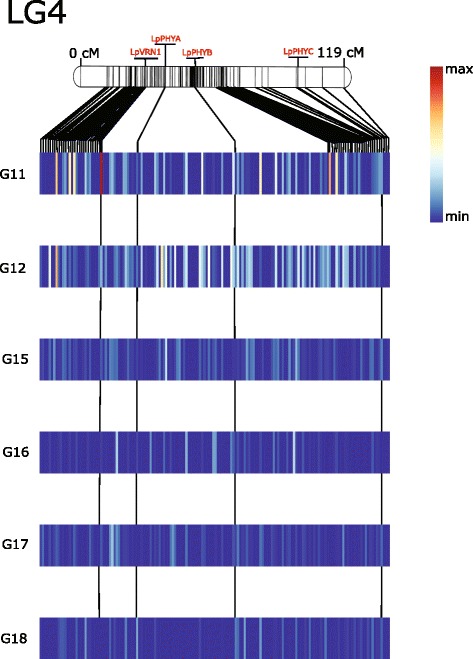
Heatmap illustrates regions associated with heading over six full-sib families in perennial ryegrass LG4. A Kruskal-Wallis test was done on each marker to identify significant regions for heading. Using perennial ryegrass genome zipper [26, 30] putative gene order for markers on LG4 was identified. These data was used to construct the heatmap for each family. Ryegrass transcriptome linkage map upon which GenomeZipper was based is used as reference to construct LG4 [26, 27] and placed above the heatmap. Each bar in the heatmap represents region between two genetic markers from the linkage map. The median Kruskal-Wallis test statistic was calculated for bins represented by gaps between markers on the genetic linkage map and used to construct the heatmap. Putative orthologs of LpVRN1, LpPHYA, LpPHYB and LpPHYC, were identified in the phylogenetic analysis and placed onto LG4 using genetic positions from genome zipper. The genetic positions of these orthologs were extrapolated over the heatmap as bars. Color of the heatmap illustrates the test-statistic of the Kruskal-wallis analysis from 0 to 21

**Fig. 6:**
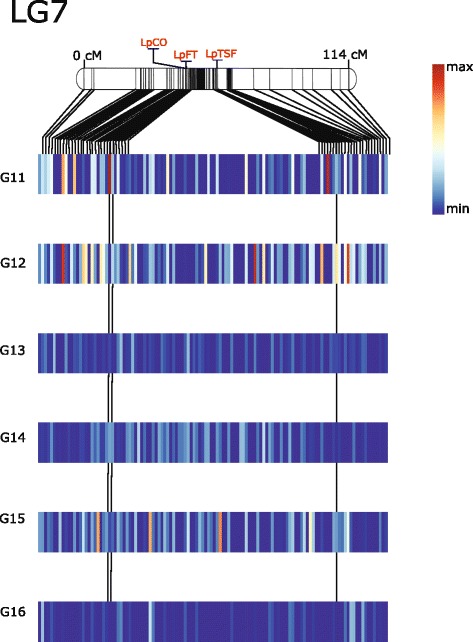
Heatmap illustrates regions associated with heading over six full-sib families in perennial ryegrass LG7. A Kruskal-Wallis test was done on each marker to identify significant regions for heading. Using perennial ryegrass genome zipper [26, 30] putative gene order for markers on LG7 was identified. These data was used to construct the heatmap for each family. Ryegrass transcriptome linkage map upon which GenomeZipper was based is used as reference to construct LG7 [26, 27] and placed above the heatmap. Each bar in the heatmap represent region between two genetic markers from linkage map. The median Kruskal-Wallis test statistic was calculated for bins represented by gaps between markers on the genetic linkage map and used to construct the heatmap. Putative orthologs of LpCO and LpFT, were identified in the phylogenetic analysis and placed onto LG7 using genetic positions from genome zipper. The genetic positions of these orthologs were extrapolated over the heatmap. Color of the heatmap illustrates the test-statistic of the Kruskal-wallis analysis from 0 to 12

The original genotypes used in the pair-crosses that generated the six full-sib families were not available for genotyping. We redid the SNP calling on each full-sib family in isolation, and filtered out variants with a minor allele frequency of less than 10 %. We only selected SNPs that were segregating in a 1:1 ratio, corresponding to sites that were homozygous in parent one and heterozygous in parent two. This was done because there are only 60 genotypes present in each full-sib family, and so any markers that segregate into more than two marker classes would have a limited number of individuals in each class. A Kruskal-Wallis test was performed on each marker to identify if they were significantly associated with heading date. We then used the perennial ryegrass GenomeZipper [26, 30] to generate a putative order for the markers according to the linkage map upon which the GenomeZipper is based. These data were used to generate heatmaps for each linkage group showing the Kruskal-Wallis test statistic (Figs. 4, 5, 6 and Additional files 2, 3, 4 and 5).

**Fig. 7:**
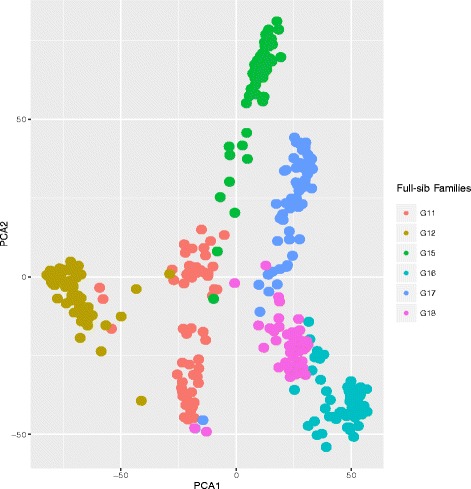
Principal component analysis (PCA) of 360 perennial ryegrass individuals, genotyped using 51,846 SNPs. The first two principal components explained 14.8 % of total variation. Components are colored according to family (color coding is listed in figure legend)

In general the strongest marker-trait associations were identified in the families G11 and G12, particularly on LG4 and LG7 (Figs. 5 and 6). The three families G15, G16, and G17 were all the result of crosses between late heading plants, and these three full-sib families showed the smallest range in days to heading (Fig. 1). Only G11 was from a cross between an early and late heading populations (Table 1). The PCA shows a separation according to the categorization of parental days to heading on the first principal component, which accounts for 7.8 % of the variation. The two full-sib families involving crosses between parents falling into different heading categories (G11 and G12) are separated from the others on PC1 (Fig. 7). We identified many markers associated with days to heading, particularly on LG4 and LG7 (Figs. 5 and 6). This was not too surprising, considering that many studies in experimental cross-populations have identified large effect QTL on the same linkage groups [9, 15–24].

G18 was the only family that showed a large amount of variation for the aftermath heading. Single marker analysis identified markers significantly associated with aftermath heading anchored onto different LGs using the GenomeZipper (Table 3). In particular we identified markers in five scaffolds anchored to LG6 in a region covering 35.9 to 56.0 cM (Table 3). We also identified markers in two scaffolds on LG2 at 80.4 and 84.2 cM, and markers in two scaffolds anchored to LG1 at 31.5 and 31.2 cM. The recent release of an annotated draft assembly of the perennial ryegrass genome [30] enables us to identify putative orthologs of key heading genes from model species. Using the GenomeZipper we can locate these on the genetic map and relate them to the marker-trait associations identified above.

**Table 3:** Single marker analysis for aftermath heading

Scaffold	Position	Test statistic	*p*-value	*q*-value	LG	cM
7674	27953	16.05	6.15x 10^−05^	0.045	1	57.5
7094	37030	15.46	8.39x 10^−05^	0.045	3	60.4
32847	126	14.82	0.00011	0.045	-	-
9166	29048	14.26	0.00015	0.045	3	28.8
4946	43369	13.69	0.00021	0.045	4	0
4946	43375	13.69	0.00021	0.045	4	0
9706	5583	13.24	0.00027	0.045	-	-
444	103726	13.08	0.00029	0.045	2	84.2
3234	46150	12.09	0.00032	0.045	-	-
444	103736	12.69	0.00036	0.045	2	84.2
444	103741	12.69	0.00036	0.045	2	84.2
13778	13753	12.69	0.00036	0.045	-	-
14987	7361	12.56	0.00039	0.045	-	-
8926	31874	12.55	0.00039	0.045	6	40.8
8309	12264	12.51	0.00040	0.045	1	31.5
8309	12267	12.51	0.00040	0.045	1	31.5
4418	6989	12.43	0.00042	0.045	1	31.2
1397	62074	12.41	0.00042	0.045	4	34.0
4165	42625	12.31	0.00044	0.045	6	55.2
9166	29013	12.23	0.00046	0.045	3	28.8
9166	29061	12.23	0.00045	0.045	3	28.8
4418	6933	12.18	0.00048	0.045	1	31.2
8672	34875	12.11	0.00050	0.045	-	-
16758	8509	12.03	0.00052	0.045	5	26.4
9159	27424	11.91	0.00055	0.045	6	44.5
500	65053	11.63	0.00064	0.045	6	35.9
1264	86342	11.63	0.00064	0.045	-	-
444	103718	11.58	0.00066	0.045	2	84.2
444	103742	11.58	0.00066	0.045	2	84.2
19325	5681	11.52	0.00068	0.045	1	13.0
5444	49105	11.51	0.00068	0.045	1	17.2
498	2811	11.51	0.00069	0.045	3	41.8
498	2853	11.51	0.00069	0.045	3	41.8
5379	10675	11.41	0.00072	0.046	3	28.8
4265	21824	11.33	0.00076	0.047	6	56.0
14987	7423	11.15	0.00084	0.048	4	45.3
9529	16294	11.11	0.00085	0.048	1	13.4
1180	59276	11.08	0.00087	0.048	2	80.4
6377	12180	11.07	0.00087	0.048	1	17.2
44	244288	11.02	0.00089	0.048	4	66.1
130	8254	10.83	0.00099	0.050	4	79.01
898	54740	10.80	0.0010	0.050	3	50.3
898	54774	10.80	0.0010	0.050	3	50.3
444	103617	10.76	0.0010	0.050	2	84.2
444	103552	10.69	0.0010	0.051	2	84.2
762	70380	10.67	0.0010	0.051	3	40.8
4165	18375	10.49	0.0011	0.051	6	55.2
441	136238	10.42	0.0012	0.051	4	45.3
441	136239	10.49	0.0012	0.051	4	45.3
9861	5433	10.40	0.0012	0.051	4	27.9

### Identifying putative orthologs of key heading genes

**Fig. 8:**
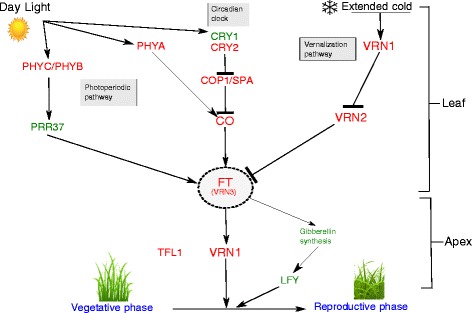
Schematic view of genetic pathway controlling heading. Genes promoting heading were shown by arrows and genes acting as repressor shown as lines with bars. External factors like day light and extended cold periods were represented with respective symbols. Pathways were mentioned in grey boxes and genes shown in red were considered as key regulators in heading

Work in model species has identified various genetic pathways controlling heading date (Fig. 8) [31]. Key genes acting within these pathways have also been characterized. We used the perennial ryegrass genome [30] to identify putative perennial ryegrass orthologs to these regulators of heading date. We used protein sequences from *Arabidopsis*, barley and rice as queries (Table 4) to search the perennial ryegrass protein set, and protein sets from *Arabidopsis thaliana* [32], *Brachypodium distachyon* [33], *Hordeum vulgare* [34], *Zea mays* [35], *Sorghum bicolor* [36], and *Oryza sativa* [37]. Only matches with a minimum query coverage of 60 % and a minimum identity of 50 % were retained for further analysis. The proteins were aligned with an alignment program MUSCLE and phylogenetic trees were built for each of 18 candidate genes. Using phylogenetic trees is the preferred method to establish orthology relationships [38]. Using this approach we were able to identify putative perennial ryegrass orthologs to eleven of these genes (Table 4). We also queried the perennial ryegrass GenomeZipper to identify putative locations for these genes on the genetic map, and relate the locations to markers we anchored as described above.

**Table 4:** Phylogenetic relationships of candidate genes involved in heading

Query candidate	Species	Protein near query	Position on Zipper	Genetic position on Zipper	Phylogenetic tree
TFL1	Arabidopsis	ms_821 |ref0016245	LG2-79.8cM	LG5-27.5cM	Figure 9
FT	Arabidopsis	ms_13332 |ref0029013	LG7-43.6cM	LG7-57.3cM	Figure 9
CRY2	Arabidopsis	ms_4185 |ref0010917	LG6-52.5cM	LG6-52.5cM	Additional file 7
GI	Arabidopsis	ms_1276 |ref0038679	LG3-29.6cM	NA	Additional file 11
PHYA	Arabidopsis	ms_13514 |ref0035067	LG4-38.5cM	LG4-38.5cM	Additional file 6
PHYB	Arabidopsis	ms_4484 |ref0039062	LG4-51.0cM	LG4-51.0cM	Additional file 6
PHYC	Arabidopsis	ms_2801 |ref0025790	LG4-98.2cM	LG4-98.2cM	Additional file 6
PRR37	Rice	ms_13366 |ref0021945	LG2-12.4cM	NA	Additional file 8
SOC1	Arabidopsis	ms_6002 |ref0025562	LG6-0cM	NA	Additional file 9
VRN1	Barley	ms_312 |ref0002704	LG4-31.4cM	LG4-31.4Cm	Additional file 12
CO	Rice	ms_5059 |ref001989	LG7-43.5cM	LG7-42.7cM	Additional file 10

We identified putative orthologs of the important photo-receptor proteins PHYA (ms_13514 |ref0035067- gene-0.0mRNA), PHYB (ms_4484 |ref0039062-gene-0.0mRNA), PHYC (ms_2801 |ref0025790-gene-0.3mRNA) and CRYTOCHROME 2 (CRY2) (ms_4185 |ref0010917-gene-0.1mRNA) (Fig. 8) (Additional file 6 and 7) and located these on the genetic map via the GenomeZipper (Fig. 5 and Additional file 5). The three Phytochromes, A, B, and C are anchored onto LG4 at different locations. All three are in locations where markers significantly associated with days to heading in one or more full-sib families. CRY2 is located on LG6 at 52.5 cM, in a region where we identified a cluster of markers between 35.9 and 56 cM that were associated with aftermath heading in G18 (Table 3). We also identified putative orthologs to PSEUDO RESPONSE REGULATOR PROTEIN 37 (PRR37) (ms_13366 |ref0021945-gene-0.0mRNA) and SUPRESSOR OF OVEREXPRESSION OF CO 1 (SOC1) (ms_6002 |ref0025562-gene-0.0mRNA) that play important roles in the central circadian clock (Additional file 8 and 9) (Fig. 4). Another important photoperiodic pathway gene is CO, which directly regulates the key floral activator FT (Fig. 8), and we have found a putative ortholog to CO (ms_5059 |ref0019898-gene-0.1mRNA) (Additional file 10) in perennial ryegrass that anchored onto LG7 at 43.5cM (Fig. 6).

Both the regulation of CO and stability of photoreceptors is controlled by GIGANTEA (GI) that is generally believed to be single copy, with a highly conserved role across the angiosperms [39]. We identified a putative ortholog to GI (ms_1276 |ref0038679-gene-0.4mRNA) (Additional file 11) in our analysis that anchored onto LG3 at 29.3cM [3] (Additional file 3). A scaffold with markers significantly associated with aftermath heading in G18 was anchored to LG3 at 28.8 cM. In addition to genes from the photoperiodic pathway, we identified a putative ortholog of the barley VERNALIZATION 1 (VRN1) (ms_312 |ref002704-gene-0.1mRNA) protein that is involved in the vernalization pathway (Additional file 12). The VRN1 protein is anchored on LG4 at 31.4cM (Fig. 5) in a region with markers significantly associated with days to heading. This was most evident in the full-sib family G11 that was generated from crossing early and late heading populations. It has already been shown that a dominant mutation in VRN1 promoter region is responsible for changes in growth habit of winter wheat to spring wheat [40]. The vernalisation and the photoperiod pathway influence heading by acting on the key floral pathway integrator FT (Fig. 8).

### Perennial ryegrass orthologs of FT and TFL1

**Fig. 9:**
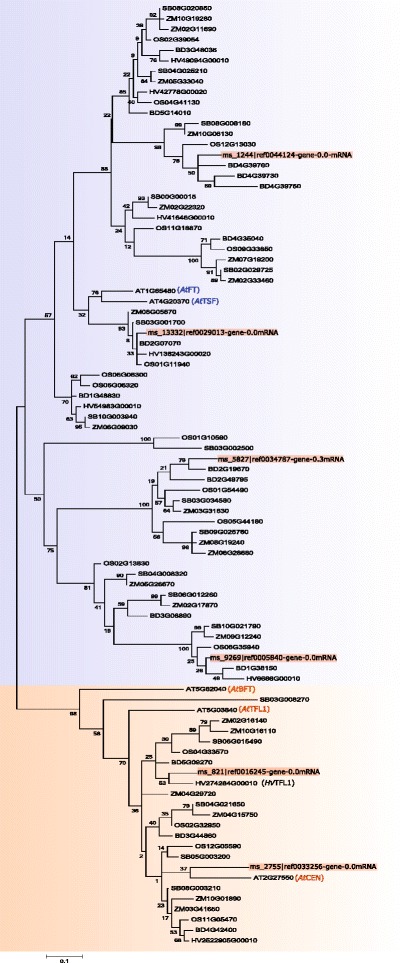
Phylogenetic analysis of FT/TFL1 gene family using *Arabidopsis* FT as query. The evolutionary history was inferred by using the Maximum Likelihood method based on the JTT matrix-based model [67]. Bootstrap values after 100 replicates were shown next to the branches. The tree is mid-point rooted, drawn to scale, with branch lengths proportional to the number of substitutions per site. The analysis involved 90 amino acid sequences. All positions containing gaps and missing data were eliminated. There were a total of 83 positions in the final dataset. Evolutionary analyses were conducted in MEGA 6.06 [66]. All the associated *Lolium* proteins are in red and *Arabidopsis* proteins were highlighted

Floral transition is controlled by FLOWERING LOCUS T (FT) and TERMINAL FLOWERING 1 (TFL1), which are genes that have functionally diverged from a common ancestor MOTHER OF FT AND TFL1 (MFT) [41]. FT promotes heading whereas TFL1 represses heading. In *Arabidopsis* the FT/TFL1 gene family consists of six members: FT, TFL1, MFT, BROTHER OF FT (BFT), CENTRORADIALIS (CEN), and TWIN SISTER OF FT (TSF). They share high sequence similarity but do have different roles in floral transition [42]. Using the *Arabidopsis* FT protein as a query we found perennial ryegrass proteins with sequence similarity to FT/TFL1 family proteins (Fig. 9). We also identified similar proteins in *Brachypodium* and barley. A phylogenetic analysis using the maximum likelihood method divided the proteins into two distinct groups, one group with the floral inducers FT and TSF and another group with the floral inhibitors TFL1, CEN and BFT (Fig. 9) [43].

Apart from floral transition, *Arabidopsis* FT also mediates stomatal opening [44]. Likewise, TFL1 is also involved in meristematic development and perennial heading [45]. We identified a putative perennial ryegrass ortholog of FT (ms_13332 |ref0029013-gene-0.0mRNA) (Fig. 9) that was anchored to LG7 at 43.6cM, in a region with markers significantly associated with heading (Fig. 6). FT was anchored to the same genetic position (43.6cM), as a previously mapped genetic marker, LpVRN3 [26]. LpVRN3 was designed on a sequence that shared 100 % identity (alignment length of 80.4 %) with the transcript we identified as orthologus to FT. Two genes from the FT/TFL gene family have previously been mapped in perennial ryegrass [27]. Both were mapped in the same experimental population used as the backbone to the GenomeZipper. Using the available genome data we can now better identify the putative perennial ryegrass orthologs to these genes. Based on our phylogentic anlaysis, the genetic marker previously labeled as LpFT is more likely to be an ortholog of TSF (ms_9269 |ref0005840-gene-0.0mRNA) (Fig. 9). TSF is the closet sequence homologue of FT and they have overlapping roles in promoting heading, however it does have a distinct role to play under short day conditions [46]. TSF was anchored to LG7 at 57.2cM (Fig. 6) in a region with markers significantly associated with heading date, particularly in family G11.

The *Arabidopsis* floral inhibitors, TFL1, BFT and CEN were grouped in a branch separate to FT. We identified putative perennial ryegrass orthologs of TFL1 (ms_821 |ref0016245-gene-0.0mRNA) clustering with barley TFL1 (Fig. 9) that was anchored on the GenomeZipper to LG2 at 79.8cM (Fig. 4). Previously a perennial ryegrass gene with sequence homology to TFL1 was anchored to LG5 at 27.5cM using a transcriptome based genetic map [27], however, our phylogentic analysis suggests that this is more likely an ortholog of BFT. In *Arabidopsis* BFT shares highest sequence similarity to TFL1 and functions similar to TFL1 in meristematic development to repress heading [47]. Interestingly, on LG2 markers we identified in single marker analysis for aftermath heading, were located on the GenomeZipper at 80.8cM and 84.2cM. These markers were next to putative perennial ryegrass ortholog of TFL1.

In perennial ryegrass, TFL1 is characterized as a repressor of heading and a regulator of axillary meristem identity [48]. When LpTFL1 was overexpressed in *Arabidopsis*, plants displayed a delayed heading phenotype and extended vegetative growth [48]. In perennial ryegrass expression level of LpTFL1 was observed in leaves, inflorescence, roots, stem and apex. It was found that after a period of cold (primary induction), expression levels of LpTFL1 reduced, allowing plants to prepare for heading. As the day length and temperature increases (secondary induction), LpTFL1 is upregulated in the apex to promote tillering [48]. Unlike annual grasses that flower once in the season and die after seed production, perennial ryegrass continues to grow even after seed production by maintaining at least one tiller in a vegetative phase. It was shown that tillering in ryegrass is mainly controlled by spatiotemporal regulatory mechanism, by activating certain genes to repress heading in vernalized tillers [49]. Interestingly, mutations in homologues of TFL1 in rose (*RoKSN*) and woodland strawberry (*FvKSN*) (both Rosoideae members of the rose family Rosaceae) have been shown to be responsible for continuous heading phenotypes in these species [50]. The putative ortholog of TFL1 identified here, which co-locates with variants for aftermath heading, is an interesting candidate gene for further study of this important forage quality trait.

## Conclusions

In this study we did not detect any SNPs significantly associated with heading and aftermath heading in a genome-wide association analysis, most likely due to the rapid decay of LD we observed in the population. However, using single marker analysis within each full-sib family we did identify linked markers, some in regions containing putative orthologs of key heading genes. Interestingly, in a family segregating for aftermath heading, SNPs were anchored proximal to a putative ortholog of TFL1, homologues of which have recently been shown to play a key role in continuous heading/aftermath heading of some Rosaceae species [50].

## Methods

### Plant material and phenotypic data

The association population consisted of 360 individual plants from six full-sib F2 families created at Oak Park, Carlow, Ireland (60 individuals selected at random from each family) (Table 1). The parents used to create the full-sib families originated from perennial ryegrass varieties (Table 1). Plants were established in the glasshouse and then transplanted into the field in a spaced plant nursery in 2013 at Oak Park, Carlow, Ireland in two replicates. Each replicate consisted of 30 blocks with 2 individuals from each full-sib family within a block. The number of days to heading from April 1^*st*^ was monitored in 2014 and 2015 for each plant. An individual plant was considered as headed, when three or more heads had emerged from the leaf sheath. In the same population aftermath heading was visually scored in the year 2015 on a scale of 1 (no aftermath heading) to 9 (intense aftermath heading) as described in [25]. Using the R package lme4 [28] variance components were estimated for heading date using genotype as a random effect and year as fixed effect. Conditional means were calculated and used for subsequent analysis.

### Genotyping full-sib families

We used a genotyping-by-sequencing approach that followed the protocol developed by Elshire et al. [51]. Briefly, genomic DNA was isolated from each individual, digested with ApeKI, samples were grouped into libraries, amplified, and sequenced on an Illumina HiSeq 2000. After sequencing, adaptor contamination was removed with Scythe [52] with a prior contamination rate set to 0.40. Sickle [53] was used to trim reads when the average quality score in a sliding window (of 20 bp) fell below a phred score of 20, and reads shorter than 40bp were discarded. The reads were demultiplexed using sabre [54] and data from each sample was aligned to the perennial ryegrass reference genome [30] using BWA [55]. The Genome Analysis Tool Kit (GATK) [56] was used to identify putative variants across the full-sib families, and also within each full-sib family. Only genotype calls with a phred score of 30 (GQ, Genotype Quality), and only variant sites with a mean mapping quality of 30 were retained. In the case of the SNP set across all full-sib families, we used a minimum minor allele frequency threshold of 5 % (Additional file 13). When identifying an SNP set within each full-sib family we used a minimum minor allele frequency threshold of 10 %.

### Genome wide association and linkage disequilibrium (LD) analysis

A mixed linear model implemented in the R package GAPIT (Genomic Association and Prediction Integrated Tool) [57] was used to perform an association analysis. The mixed model accounts for population structure and family relatedness using principal component analysis (PCA) and a kinship matrix calculated by GAPIT with available input genotypic data. To account for multiple testing during association analysis, false discovery rate (FDR) [58] and Bonferroni correction (0.05/51864) [59] with an *α* level of 0.05 was used setting a threshold at 9.8x10 ^−7^. To assess the extent of LD across the full-sib populations we identified SNPs located within a single genomic scaffold, and calculated the inter SNP distance and the squared correlation of the allele counts in Plink 1.9 [60], based on the maximum likelihood solution to the cubic Eq. [61].

### Pipeline for single marker analysis

A SNP panel was developed for each full-sib family, using a 10 % of minor allele frequency, and subsequent analyis was performed on each of the six full sib families independently. SNPs segregating in a 1:1 ratio were selected, that is homozygous in parent one and heterozygous in parent two. A *X*^2^ test was used to eliminate SNPs that deviated significantly from a 1:1 segregation. We then performed a Kruskal-Wallis test using R [62] on each marker to check for association with heading date. Using the GenomeZipper [26, 30] we established a putative order for these markers along the genetic map. The median Kruskal-Wallis test statistic was calculated for bins represented by gaps between markers on the genetic linkage map.

### Protein datasets and phylogenetic analysis

The query proteins for key heading genes was obtained from *Arabidopsis thaliana*, rice and barley using the uniport database [63] (Table 4). The complete protein sets from perennial ryegrass [30], *Arabidopsis* [32], *Brachypodium* [33], barley [34], rice [37], *Sorghum* [36] and maize [35] were gathered from PLAZA 3.0 [64] and combined into single file to build a BLASTp database. Using each query we performed a BLASTp with an evalue of 10e-10 and parsed the results for hits with at least 60 % coverage and 50 % identity. The sequences were aligned using MUSCLE [65], an alignment program implemented in MEGA 6.06 [66]. The phylogentic analysis was carried out using the Maximum Likelihood method based on the JTT matrix-based model in MEGA 6.06 [66, 67]. Bootstrap values after 100 replicates are shown next to the branches. Initial tree(s) for the heuristic search were obtained automatically by applying Neighbor-Join and BioNJ algorithms to a matrix of pairwise distances estimated using a JTT model, and then selecting the topology with superior log likelihood value. The tree is mid-point rooted, drawn to scale, with branch lengths measured in the number of substitutions per site.

## Abbreviations

GWAS, genome wide association study; QTL, quantitative trait loci; LD, linkage disequilibrium; LG, linkage group

## Additional files

Additional file 1
**Figure S1.** Scatter plot for heading vs aftermath heading. Linear regression was done using aftermath heading as response variable and heading as explantory variable for family G18. Early heading genotypes showed tendency of higher aftermath heading. (PDF 10 kb)

Additional file 2
**Figure S2.** Heatmap of perennial ryegrass LG1 over six full-sib families. A Kruskal-Wallis test was performed on each marker to identify significant regions for heading. Using the perennial ryegrass genome zipper [26, 30] we identified a putative gene order for markers on LG1. These data were used to construct the heatmap for each family. A perennial ryegrass transcriptome-based genetic linkage map upon which GenomeZipper was based was used as reference to construct LG1 [26, 27] and placed above the heatmap. Each bar in the heatmap represents region between two genetic markers from the linkage map. The median Kruskal-Wallis test statistic was calculated for markers binned between markers on the genetic linkage map and used to construct the heatmap. Color of the heatmap illustrates the test-statistic of the Kruskal-wallis analysis. (PDF 31 kb)

Additional file 3
**Figure S3.** Heatmap of perennial ryegrass LG3 over six full-sib families. A Kruskal-Wallis test was performed on each marker to identify significant regions for heading. Using the perennial ryegrass genome zipper [26, 30] we identified a putative gene order for markers on LG3. These data were used to construct the heatmap for each family. A perennial ryegrass transcriptome-based genetic linkage map upon which GenomeZipper was based was used as reference to construct LG3 [26, 27] and placed above the heatmap. Each bar in the heatmap represents region between two genetic markers from the linkage map. The median Kruskal-Wallis test statistic was calculated for markers binned between markers on the genetic linkage map and used to construct the heatmap. Putative ortholog of LpGI, was identified in the phylogenetic analysis and placed onto LG3 using genetic positions from genome zipper. The genetic positions of these orthologs were extrapolated over the heatmap. Color of the heatmap illustrates the test-statistic of the Kruskal-wallis analysis. (PDF 26 kb)

Additional file 4
**Figure S4.** Heatmap of perennial ryegrass LG5 over six full-sib families. A Kruskal-Wallis test was performed on each marker to identify significant regions for heading. Using the perennial ryegrass genome zipper [26, 30] we identified a putative gene order for markers on LG5. These data were used to construct the heatmap for each family. A perennial ryegrass transcriptome-based genetic linkage map upon which GenomeZipper was based was used as reference to construct LG5 [26, 27] and placed above the heatmap. Each bar in the heatmap represents region between two genetic markers from the linkage map. The median Kruskal-Wallis test statistic was calculated for markers binned between markers on the genetic linkage map and used to construct the heatmap. Color of the heatmap illustrates the test-statistic of the Kruskal-wallis analysis. (PDF 32 kb)

Additional file 5
**Figure S5.** Heatmap of perennial ryegrass LG6 over six full-sib families. A Kruskal-Wallis test was performed on each marker to identify significant regions for heading. Using the perennial ryegrass genome zipper [26, 30] we identified a putative gene order for markers on LG6. These data were used to construct the heatmap for each family. A perennial ryegrass transcriptome-based genetic linkage map upon which GenomeZipper was based was used as reference to construct LG6 [26, 27] and placed above the heatmap. Each bar in the heatmap represents region between two genetic markers from the linkage map. The median Kruskal-Wallis test statistic was calculated for markers binned between markers on the genetic linkage map and used to construct the heatmap. Putative ortholog of LpCRY2 was identified in the phylogenetic analysis and placed onto LG6 using genetic positions from genome zipper. The genetic positions of these orthologs were extrapolated over the heatmap. Color of the heatmap illustrates the test-statistic of the Kruskal-wallis analysis. (PDF 35 kb)

Additional file 6
**Figure S6.** Phylogenetic tree of candidate heading genes PHYA, PHYB and PHYC. The evolutionary history was inferred by using the Maximum Likelihood method based on the JTT matrix-based model [67]. The tree is mid-point rooted, drawn to scale, with branch lengths proportional to the number of substitutions per site. All positions containing gaps and missing data were eliminated. Evolutionary analyses were conducted in MEGA 6.06 [66]. All the associated *Lolium* and *Arabidopsis* proteins were highlighted. (PDF 59 kb)

Additional file 7
**Figure S7.** Phylogenetic tree of candidate heading gene CRY2. The evolutionary history was inferred by using the Maximum Likelihood method based on the JTT matrix-based model [67]. The tree is mid-point rooted, drawn to scale, with branch lengths proportional to the number of substitutions per site. All positions containing gaps and missing data were eliminated. Evolutionary analyses were conducted in MEGA 6.06 [66]. All the associated *Lolium* and *Arabidopsis* proteins were highlighted. (PDF 40 kb)

Additional file 8
**Figure S8.** Phylogenetic tree of candidate heading gene PRR37. The evolutionary history was inferred by using the Maximum Likelihood method based on the JTT matrix-based model [67]. The tree is mid-point rooted, drawn to scale, with branch lengths proportional to the number of substitutions per site. All positions containing gaps and missing data were eliminated. Evolutionary analyses were conducted in MEGA 6.06 [66]. All the associated *Lolium* and rice proteins were highlighted. (PDF 38 kb)

Additional file 9
**Figure S9.** Phylogenetic tree of candidate heading gene SOC1. The evolutionary history was inferred by using the Maximum Likelihood method based on the JTT matrix-based model [67]. The tree is mid-point rooted, drawn to scale, with branch lengths proportional to the number of substitutions per site. All positions containing gaps and missing data were eliminated. Evolutionary analyses were conducted in MEGA 6.06 [66]. All the associated *Lolium* and *Arabidopsis* proteins were highlighted. (PDF 42 kb)

Additional file 10
**Figure S10.** Phylogenetic tree of candidate heading gene CO. The evolutionary history was inferred by using the Maximum Likelihood method based on the JTT matrix-based model [67]. The tree is mid-point rooted, drawn to scale, with branch lengths proportional to the number of substitutions per site. All positions containing gaps and missing data were eliminated. Evolutionary analyses were conducted in MEGA 6.06 [66]. Associated *Lolium* and rice proteins were highlighted. (PDF 37 kb)

Additional file 11
**Figure S11.** Phylogenetic tree of candidate heading gene GI. The evolutionary history was inferred by using the Maximum Likelihood method based on the JTT matrix-based model [67]. The tree is mid-point rooted, drawn to scale, with branch lengths proportional to the number of substitutions per site. All positions containing gaps and missing data were eliminated. Evolutionary analyses were conducted in MEGA 6.06 [66]. Associated *Lolium* and *Arabidopsis* proteins were highlighted. (PDF 37 kb)

Additional file 12
**Figure S12.** Phylogenetic tree of candidate heading gene VRN1. The evolutionary history was inferred by using the Maximum Likelihood method based on the JTT matrix-based model [67]. The tree is mid-point rooted, drawn to scale, with branch lengths proportional to the number of substitutions per site. All positions containing gaps and missing data were eliminated. Evolutionary analyses were conducted in MEGA 6.06 [66]. Associated *Lolium* and barley proteins were highlighted. (PDF 40 kb)

Additional file 13
**Data set S1.** SNP positions and genotype calls. This additional data set contains SNP positions relative to the published draft genome [30]. The first column identifies the scaffold containing the SNP. The value after the final underscore identifies the SNP position within this scaffold. The second column identifies the alleles, and the remaining columns are the genotype calls for each individual. NN refers to a missing genotype. (ZIP 6379 kb)
