# Supplementary figures and images for: Markers associated with heading and aftermath heading in perennial ryegrass full-sib families
Source: BMC Plant Biol. 2016 Jul 16;16:160. doi: 10.1186/s12870-016-0844-y (PMC4947259; doi:10.1186/s12870-016-0844-y)

Adj R2 = 0.17044 Intercept = 3.9323 Slope = -0.20561 P = 0.00083216

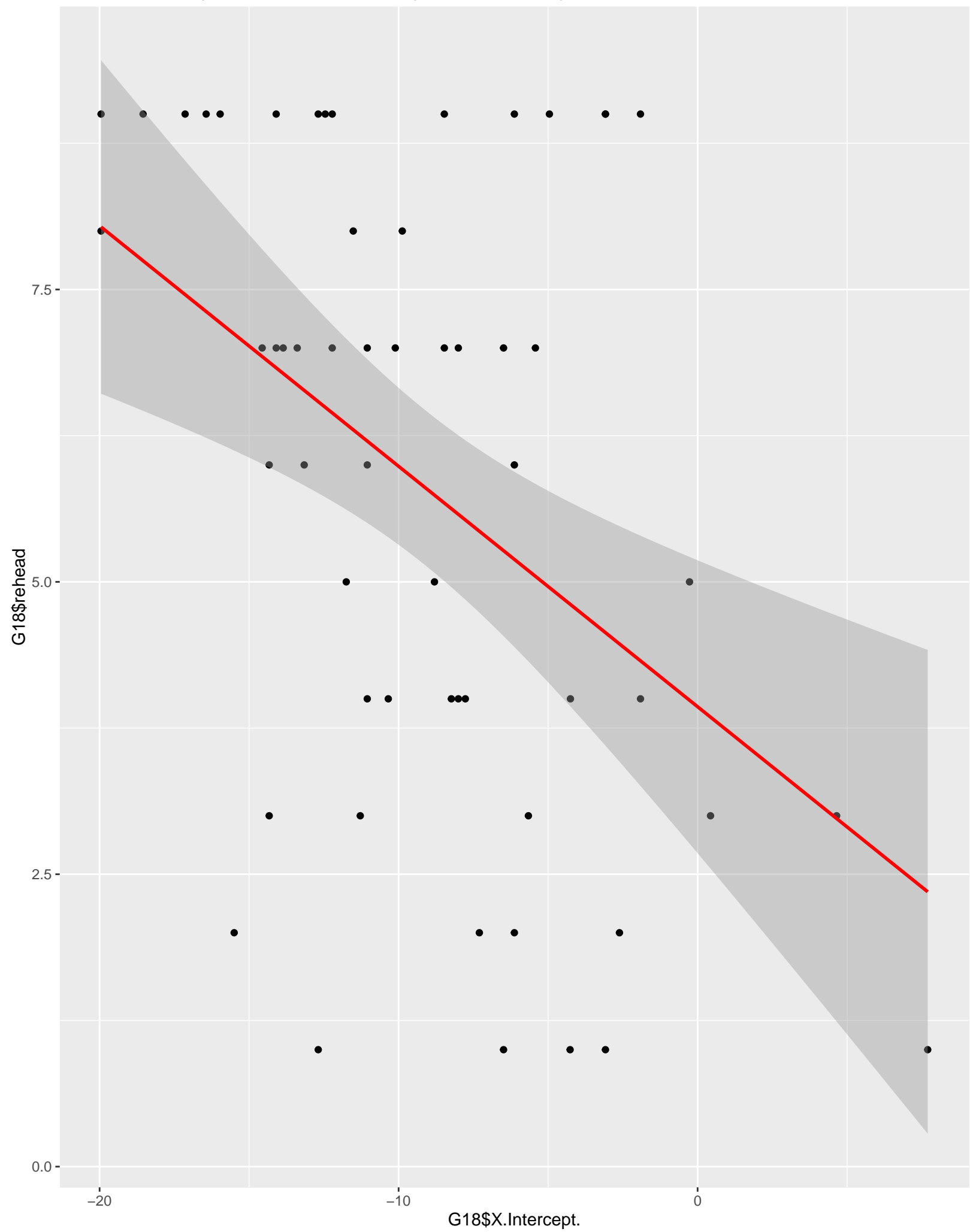

Supplement: Additional file 1 — Figure S1. Scatter plot for heading vs aftermath heading. Linear regression was done using aftermath heading as response variable and heading as explantory variable for family G18. Early heading genotypes showed tendency of higher aftermath heading. (PDF 10 kb) [file 12870_2016_844_MOESM1_ESM.pdf]

# LG1

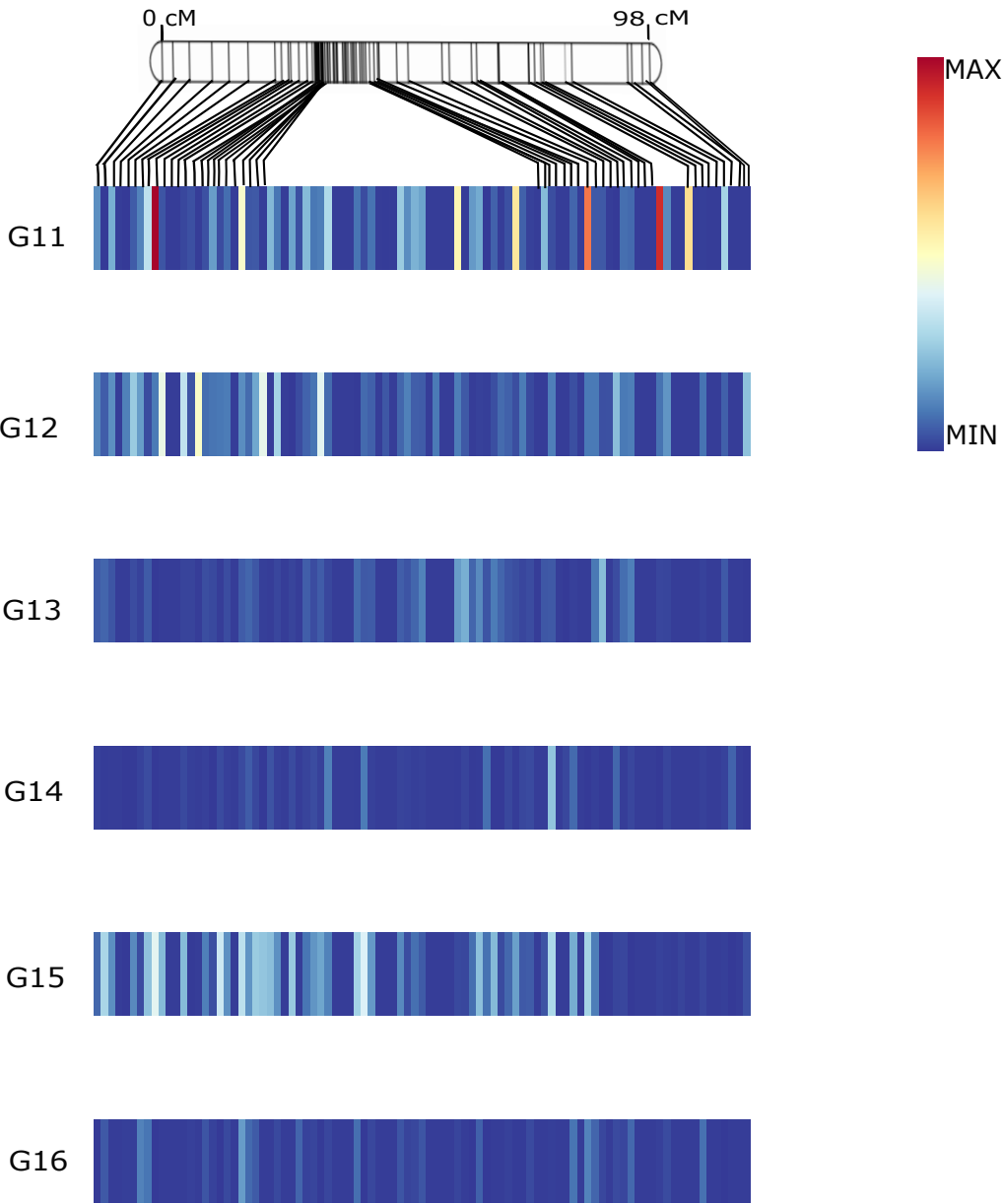

Supplement: Additional file 2 — Figure S2. Heatmap of perennial ryegrass LG1 over six full-sib families. A Kruskal-Wallis test was performed on each marker to identify significant regions for heading. Using the perennial ryegrass genome zipper [26, 30] we identified a putative gene order for markers on LG1. These data were used to construct the heatmap for each family. A perennial ryegrass transcriptome-based genetic linkage map upon which GenomeZipper was based was used as reference to construct LG1 [26, 27] and placed above the heatmap. Each bar in the heatmap represents region between two genetic markers from the linkage map. The median Kruskal-Wallis test statistic was calculated for markers binned between markers on the genetic linkage map and used to construct the heatmap. Color of the heatmap illustrates the test-statistic of the Kruskal-wallis analysis. (PDF 31 kb) [file 12870_2016_844_MOESM2_ESM.pdf]

# LG3

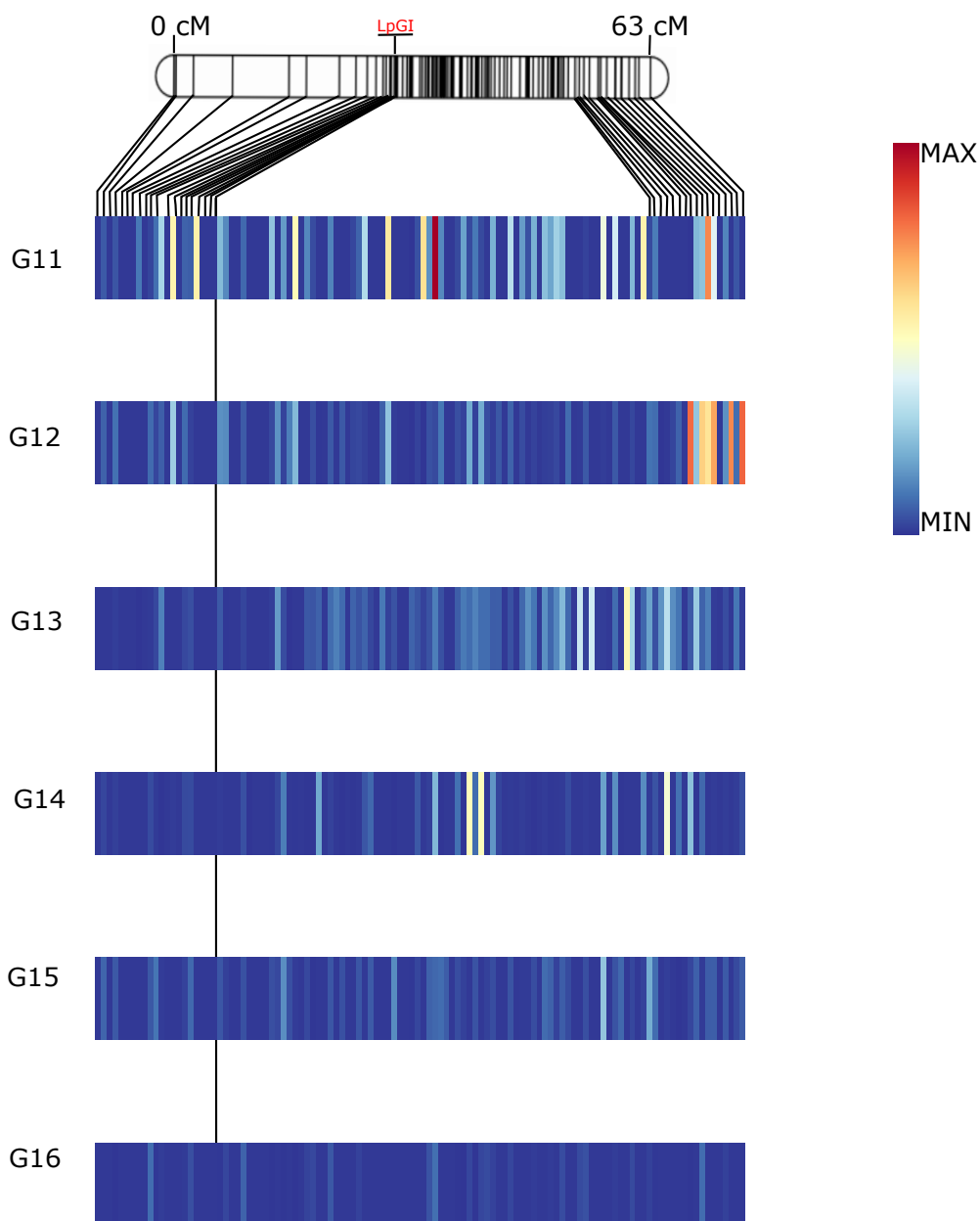

Supplement: Additional file 3 — Figure S3. Heatmap of perennial ryegrass LG3 over six full-sib families. A Kruskal-Wallis test was performed on each marker to identify significant regions for heading. Using the perennial ryegrass genome zipper [26, 30] we identified a putative gene order for markers on LG3. These data were used to construct the heatmap for each family. A perennial ryegrass transcriptome-based genetic linkage map upon which GenomeZipper was based was used as reference to construct LG3 [26, 27] and placed above the heatmap. Each bar in the heatmap represents region between two genetic markers from the linkage map. The median Kruskal-Wallis test statistic was calculated for markers binned between markers on the genetic linkage map and used to construct the heatmap. Putative ortholog of LpGI, was identified in the phylogenetic analysis and placed onto LG3 using genetic positions from genome zipper. The genetic positions of these orthologs were extrapolated over the heatmap. Color of the heatmap illustrates the test-statistic of the Kruskal-wallis analysis. (PDF 26 kb) [file 12870_2016_844_MOESM3_ESM.pdf]

# LG5

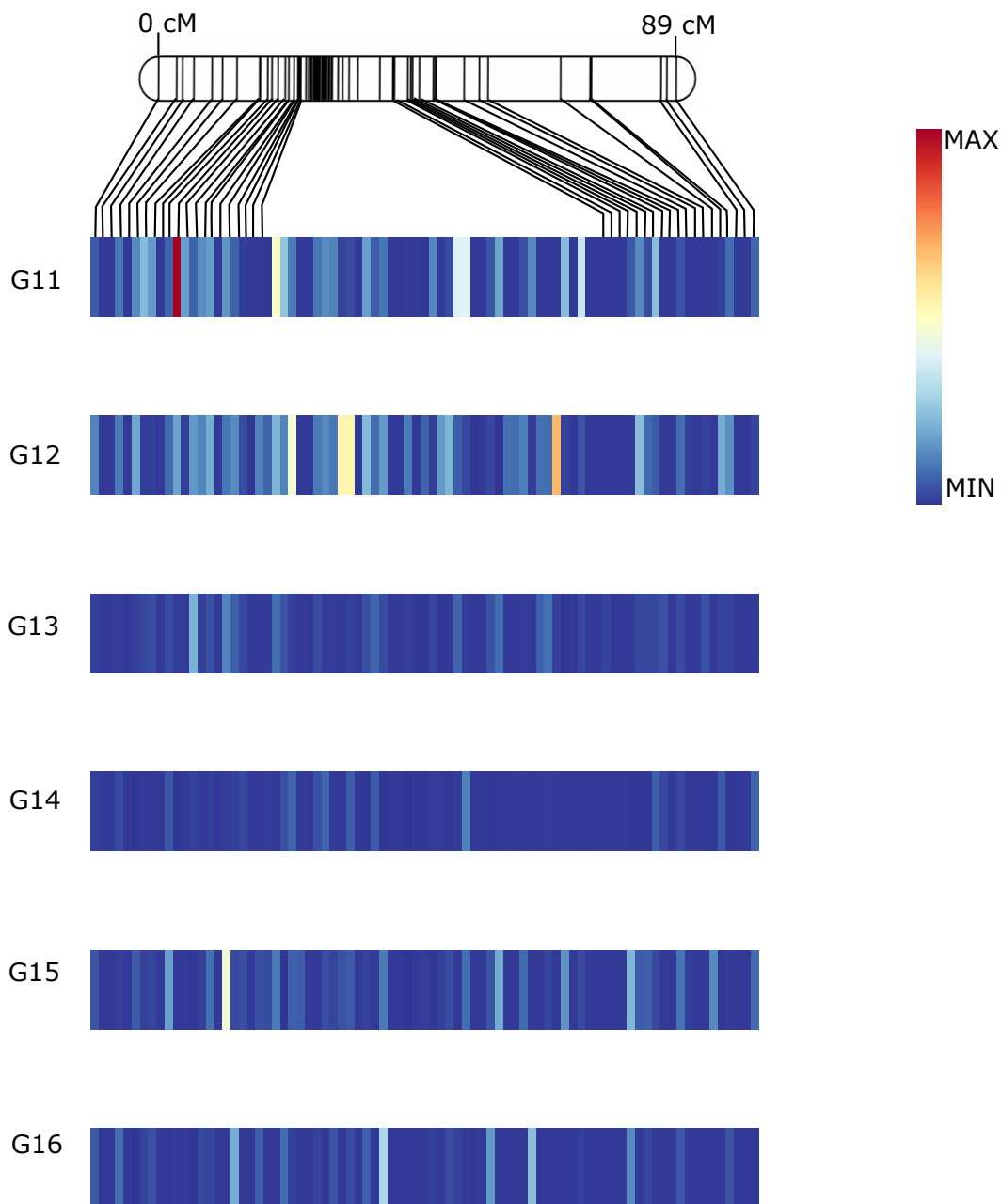

Supplement: Additional file 4 — Figure S4. Heatmap of perennial ryegrass LG5 over six full-sib families. A Kruskal-Wallis test was performed on each marker to identify significant regions for heading. Using the perennial ryegrass genome zipper [26, 30] we identified a putative gene order for markers on LG5. These data were used to construct the heatmap for each family. A perennial ryegrass transcriptome-based genetic linkage map upon which GenomeZipper was based was used as reference to construct LG5 [26, 27] and placed above the heatmap. Each bar in the heatmap represents region between two genetic markers from the linkage map. The median Kruskal-Wallis test statistic was calculated for markers binned between markers on the genetic linkage map and used to construct the heatmap. Color of the heatmap illustrates the test-statistic of the Kruskal-wallis analysis. (PDF 32 kb) [file 12870_2016_844_MOESM4_ESM.pdf]

# LG6

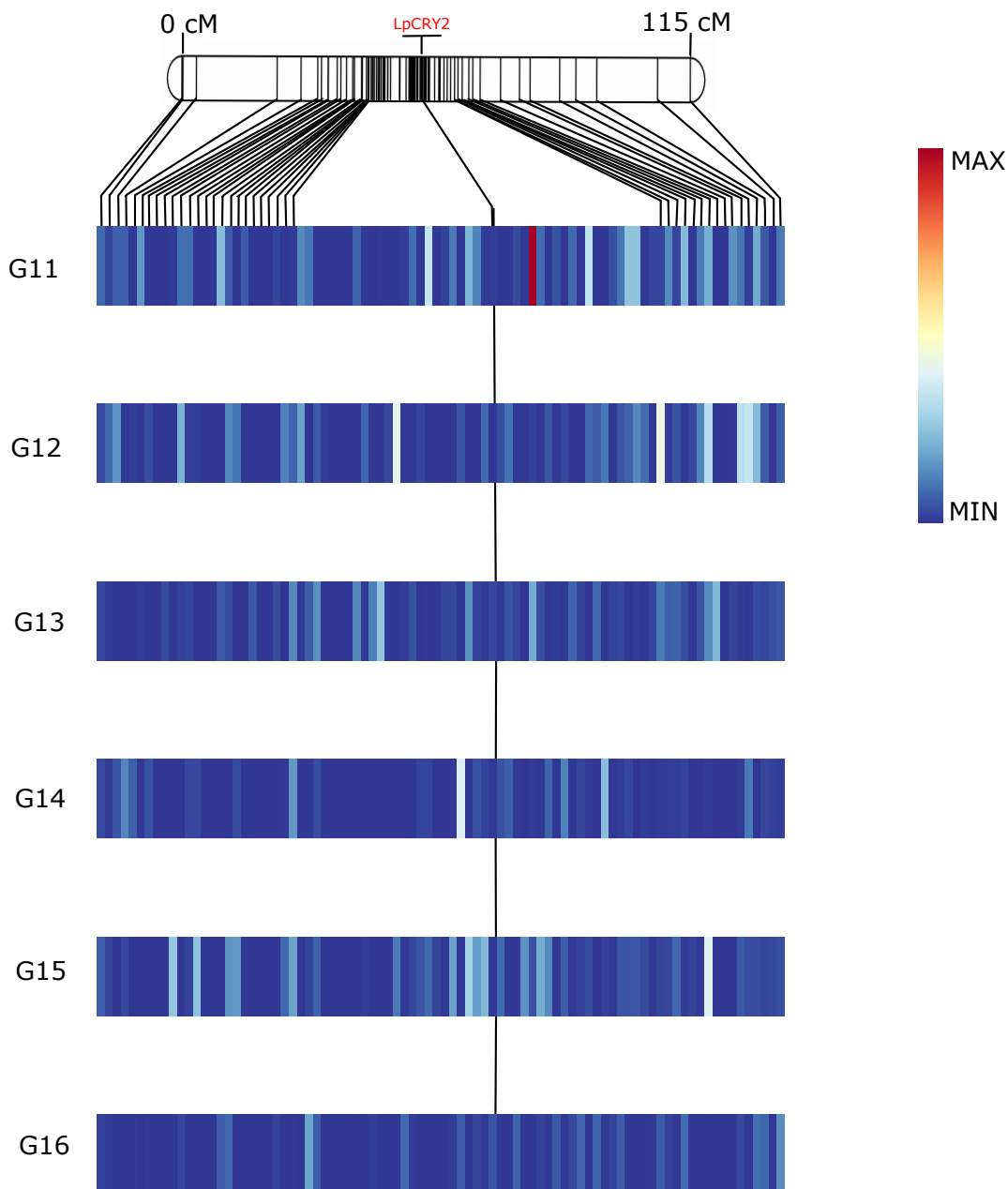

Supplement: Additional file 5 — Figure S5. Heatmap of perennial ryegrass LG6 over six full-sib families. A Kruskal-Wallis test was performed on each marker to identify significant regions for heading. Using the perennial ryegrass genome zipper [26, 30] we identified a putative gene order for markers on LG6. These data were used to construct the heatmap for each family. A perennial ryegrass transcriptome-based genetic linkage map upon which GenomeZipper was based was used as reference to construct LG6 [26, 27] and placed above the heatmap. Each bar in the heatmap represents region between two genetic markers from the linkage map. The median Kruskal-Wallis test statistic was calculated for markers binned between markers on the genetic linkage map and used to construct the heatmap. Putative ortholog of LpCRY2 was identified in the phylogenetic analysis and placed onto LG6 using genetic positions from genome zipper. The genetic positions of these orthologs were extrapolated over the heatmap. Color of the heatmap illustrates the test-statistic of the Kruskal-wallis analysis. (PDF 35 kb) [file 12870_2016_844_MOESM5_ESM.pdf]

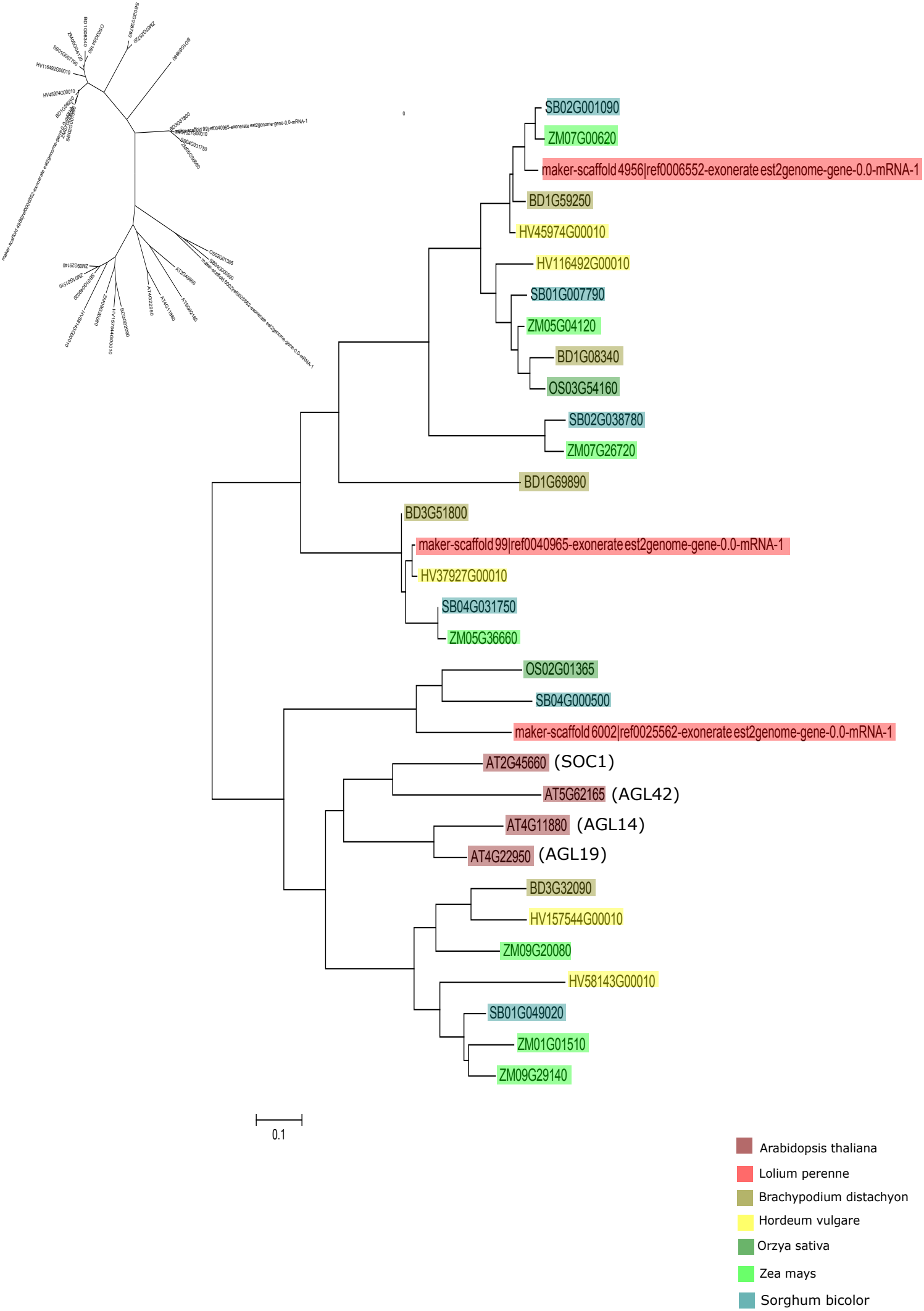

Supplement: Additional file 9 — Figure S9. Phylogenetic tree of candidate heading gene SOC1. The evolutionary history was inferred by using the Maximum Likelihood method based on the JTT matrix-based model [67]. The tree is mid-point rooted, drawn to scale, with branch lengths proportional to the number of substitutions per site. All positions containing gaps and missing data were eliminated. Evolutionary analyses were conducted in MEGA 6.06 [66]. All the associated Lolium and Arabidopsis proteins were highlighted. (PDF 42 kb) [file 12870_2016_844_MOESM9_ESM.pdf]

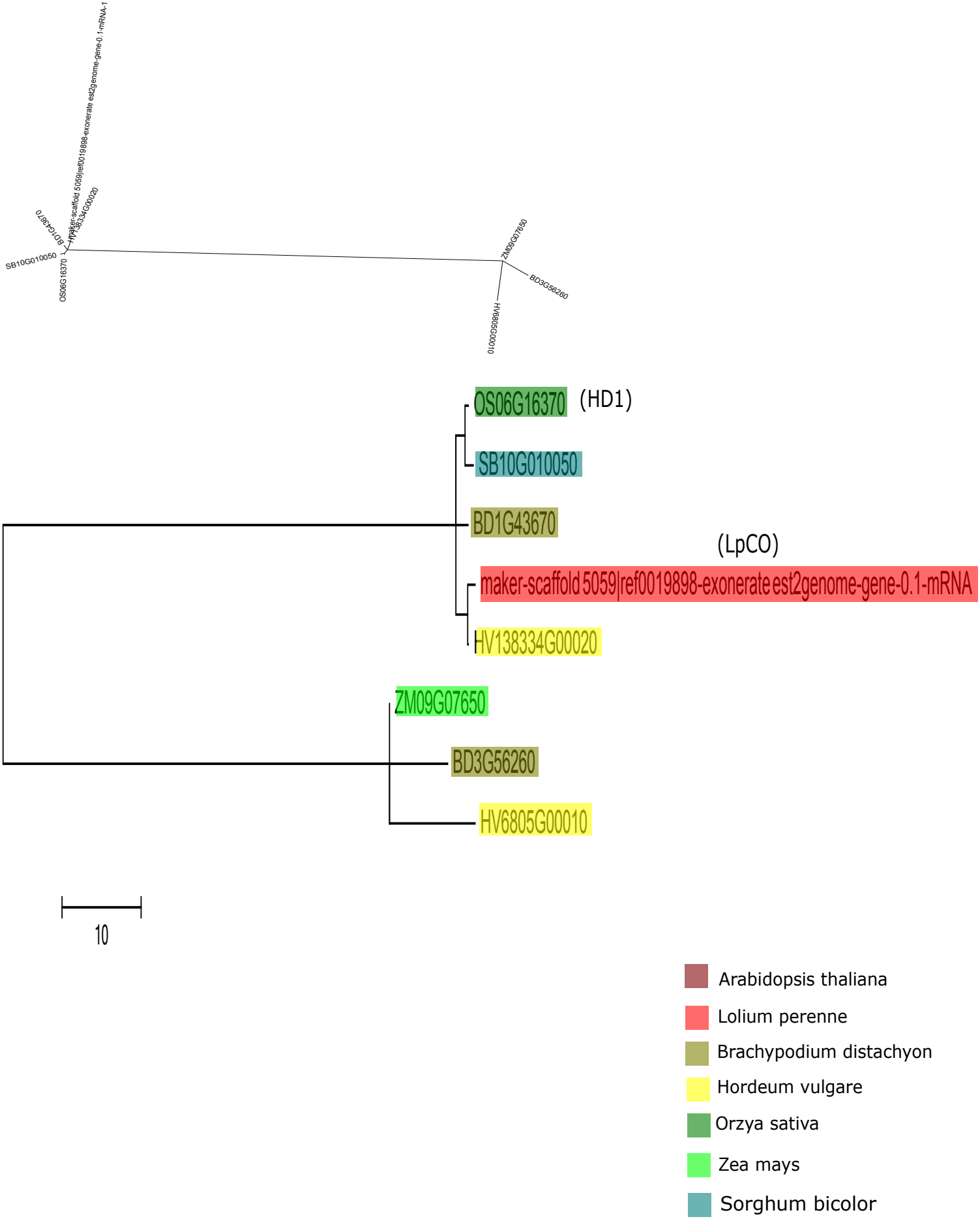

Supplement: Additional file 10 — Figure S10. Phylogenetic tree of candidate heading gene CO. The evolutionary history was inferred by using the Maximum Likelihood method based on the JTT matrix-based model [67]. The tree is mid-point rooted, drawn to scale, with branch lengths proportional to the number of substitutions per site. All positions containing gaps and missing data were eliminated. Evolutionary analyses were conducted in MEGA 6.06 [66]. Associated Lolium and rice proteins were highlighted. (PDF 37 kb) [file 12870_2016_844_MOESM10_ESM.pdf]

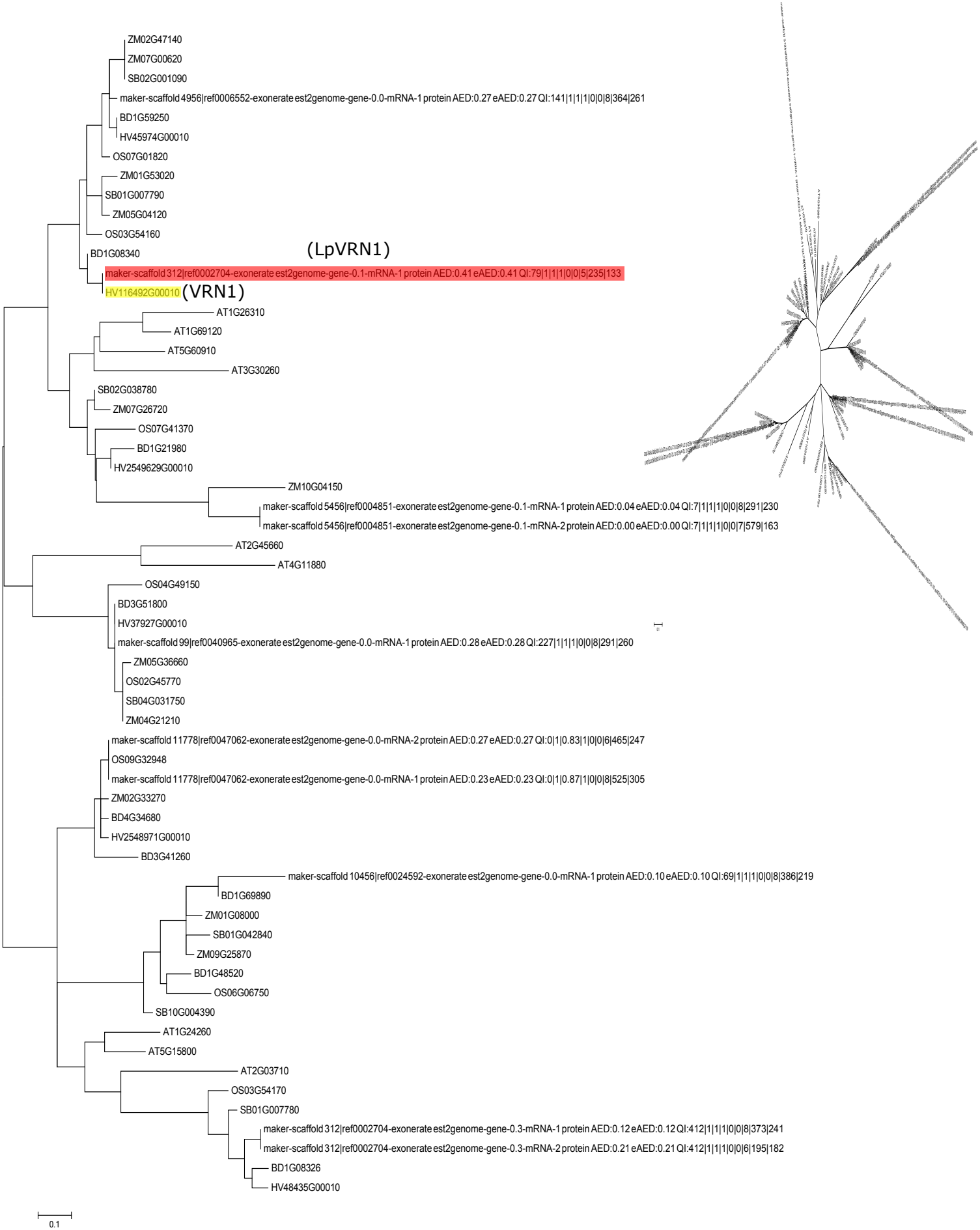

Supplement: Additional file 12 — Figure S12. Phylogenetic tree of candidate heading gene VRN1. The evolutionary history was inferred by using the Maximum Likelihood method based on the JTT matrix-based model [67]. The tree is mid-point rooted, drawn to scale, with branch lengths proportional to the number of substitutions per site. All positions containing gaps and missing data were eliminated. Evolutionary analyses were conducted in MEGA 6.06 [66]. Associated Lolium and barley proteins were highlighted. (PDF 40 kb) [file 12870_2016_844_MOESM12_ESM.pdf]
