# Supplementary material for: Markers associated with heading and aftermath heading in perennial ryegrass full-sib families
Source: BMC Plant Biol. 2016 Jul 16;16:160. doi: 10.1186/s12870-016-0844-y (PMC4947259; doi:10.1186/s12870-016-0844-y)
Supplement: Additional file 6 — Figure S6. Phylogenetic tree of candidate heading genes PHYA, PHYB and PHYC. The evolutionary history was inferred by using the Maximum Likelihood method based on the JTT matrix-based model [67]. The tree is mid-point rooted, drawn to scale, with branch lengths proportional to the number of substitutions per site. All positions containing gaps and missing data were eliminated. Evolutionary analyses were conducted in MEGA 6.06 [66]. All the associated Lolium and Arabidopsis proteins were highlighted. (PDF 59 kb) [file 12870_2016_844_MOESM6_ESM.pdf]

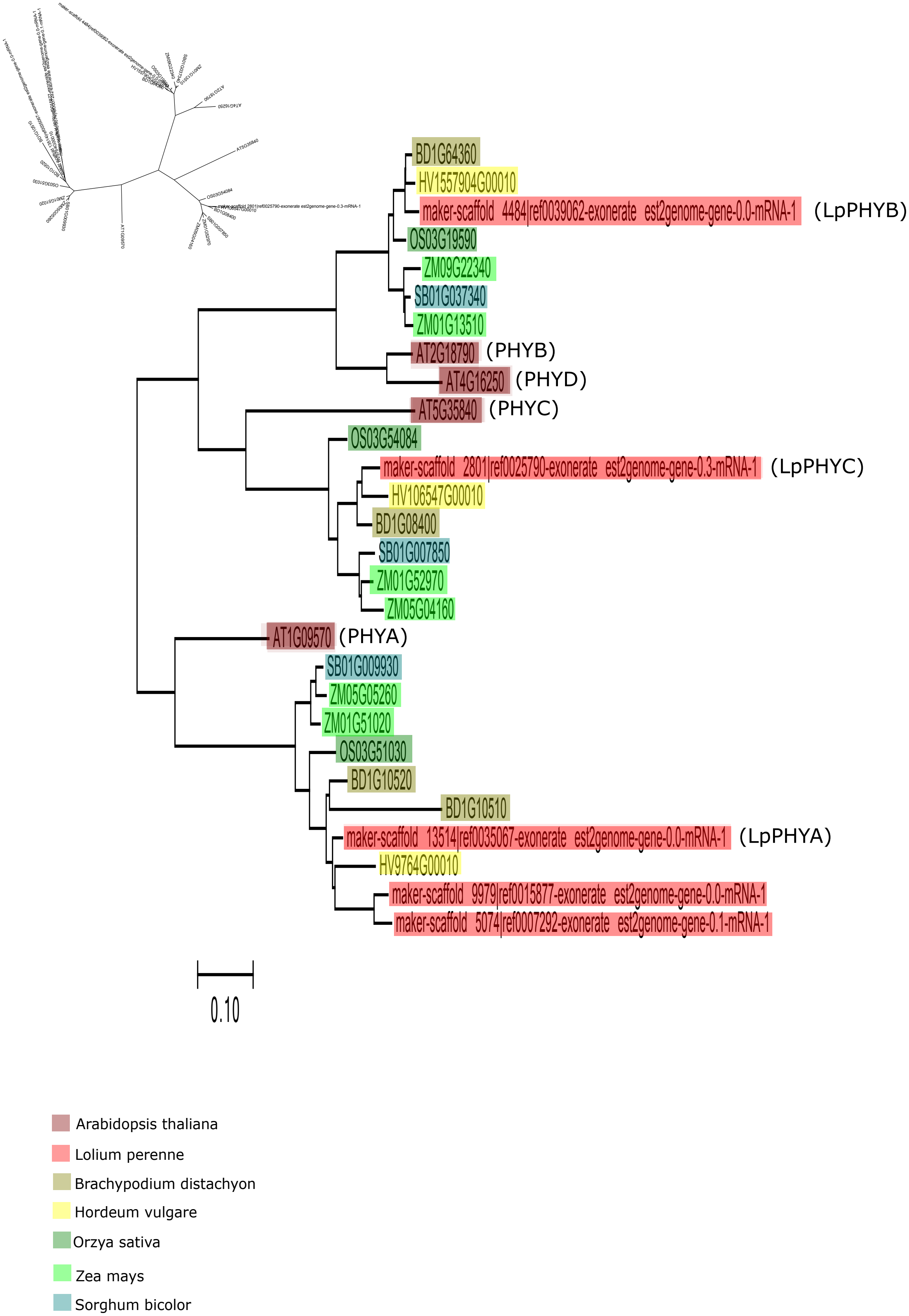
BD1G64360
HV1557904G00010
maker-scaffold 4484|ref0039062-exonerate est2genome-gene-0.0-mRNA-1
(LpPHYB)
OS03G19590
ZM09G22340
SB01G037340
ZM01G13510
AT2G18790
(PHYB)
AT4G16250
(PHYD)
AT5G35840
(PHYC)
OS03G54084
maker-scaffold 2801|ref0025790-exonerate est2genome-gene-0.3-mRNA-1
(LpPHYC)
HV106547G00010
BD1G08400
SB01G007850
ZM01G52970
ZM05G04160
AT1G09570
(PHYA)
SB01G009930
ZM05G05260
ZM01G51020
OS03G51030
BD1G10520
BD1G10510
maker-scaffold 13514|ref0035067-exonerate est2genome-gene-0.0-mRNA-1
(LpPHYA)
HV9764G00010
maker-scaffold 9979|ref0015877-exonerate est2genome-gene-0.0-mRNA-1
maker-scaffold 5074|ref0007292-exonerate est2genome-gene-0.1-mRNA-1
0.10
Arabidopsis thaliana
Lolium perenne
Brachypodium distachyon
Hordeum vulgare
Orzya sativa
Zea mays
Sorghum bicolor
