# Supplementary material for: Markers associated with heading and aftermath heading in perennial ryegrass full-sib families
Source: BMC Plant Biol. 2016 Jul 16;16:160. doi: 10.1186/s12870-016-0844-y (PMC4947259; doi:10.1186/s12870-016-0844-y)
Supplement: Additional file 7 — Figure S7. Phylogenetic tree of candidate heading gene CRY2. The evolutionary history was inferred by using the Maximum Likelihood method based on the JTT matrix-based model [67]. The tree is mid-point rooted, drawn to scale, with branch lengths proportional to the number of substitutions per site. All positions containing gaps and missing data were eliminated. Evolutionary analyses were conducted in MEGA 6.06 [66]. All the associated Lolium and Arabidopsis proteins were highlighted. (PDF 40 kb) [file 12870_2016_844_MOESM7_ESM.pdf]

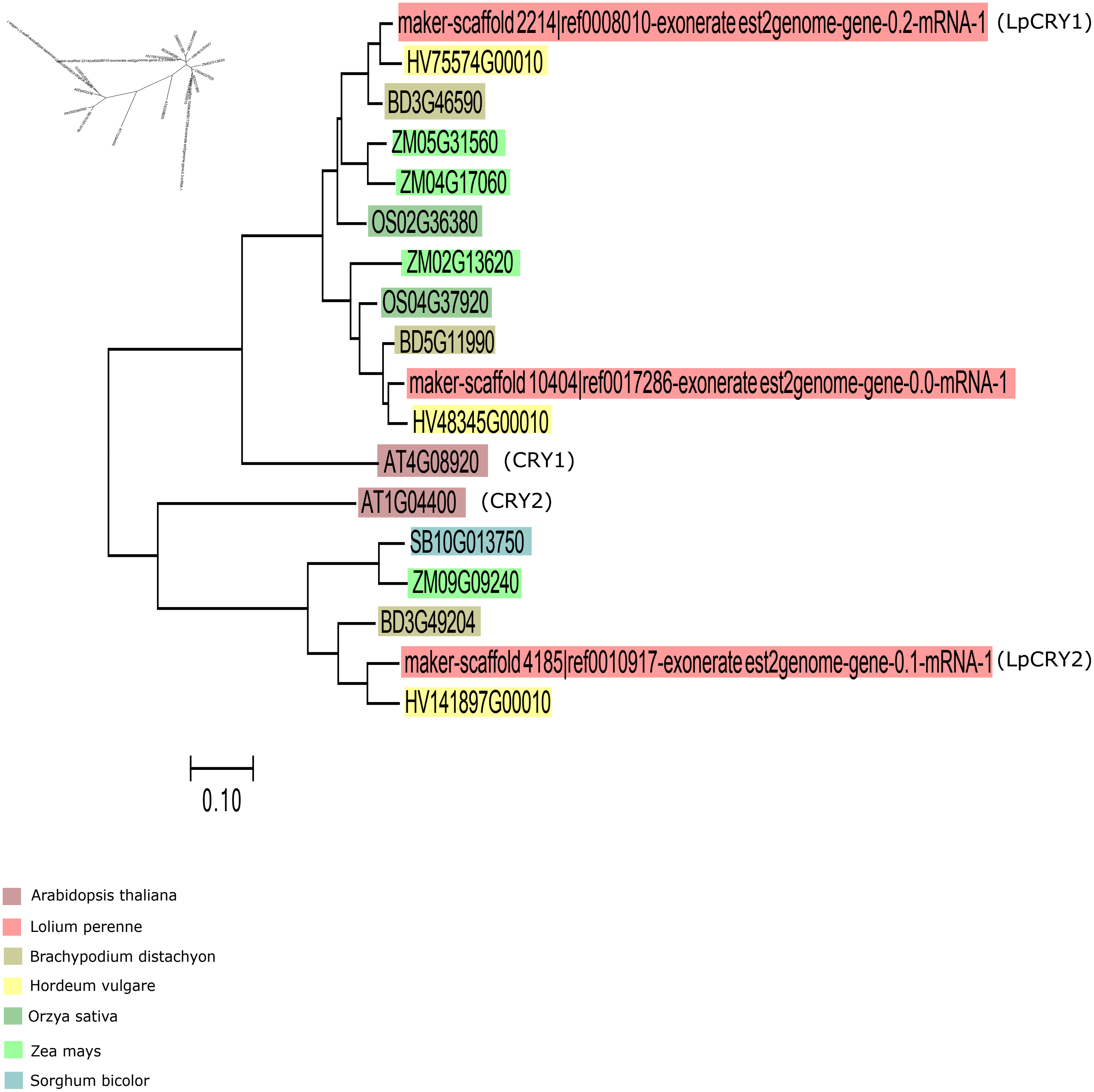

maker-scaffold 2214|ref0008010-exonerate est2genome-gene-0.2-mRNA-1 (LpCRY1)
HV75574G00010
BD3G46590
ZM05G31560
ZM04G17060
OS02G36380
ZM02G13620
OS04G37920
BD5G11990
maker-scaffold 10404|ref0017286-exonerate est2genome-gene-0.0-mRNA-1
HV48345G00010
AT4G08920 (CRY1)
AT1G04400 (CRY2)
SB10G013750
ZM09G09240
BD3G49204
maker-scaffold 4185|ref0010917-exonerate est2genome-gene-0.1-mRNA-1 (LpCRY2)
HV141897G00010
0.10
Arabidopsis thaliana
Lolium perenne
Brachypodium distachyon
Hordeum vulgare
Orzya sativa
Zea mays
Sorghum bicolor
