# Supplementary material for: Markers associated with heading and aftermath heading in perennial ryegrass full-sib families
Source: BMC Plant Biol. 2016 Jul 16;16:160. doi: 10.1186/s12870-016-0844-y (PMC4947259; doi:10.1186/s12870-016-0844-y)
Supplement: Additional file 8 — Figure S8. Phylogenetic tree of candidate heading gene PRR37. The evolutionary history was inferred by using the Maximum Likelihood method based on the JTT matrix-based model [67]. The tree is mid-point rooted, drawn to scale, with branch lengths proportional to the number of substitutions per site. All positions containing gaps and missing data were eliminated. Evolutionary analyses were conducted in MEGA 6.06 [66]. All the associated Lolium and rice proteins were highlighted. (PDF 38 kb) [file 12870_2016_844_MOESM8_ESM.pdf]

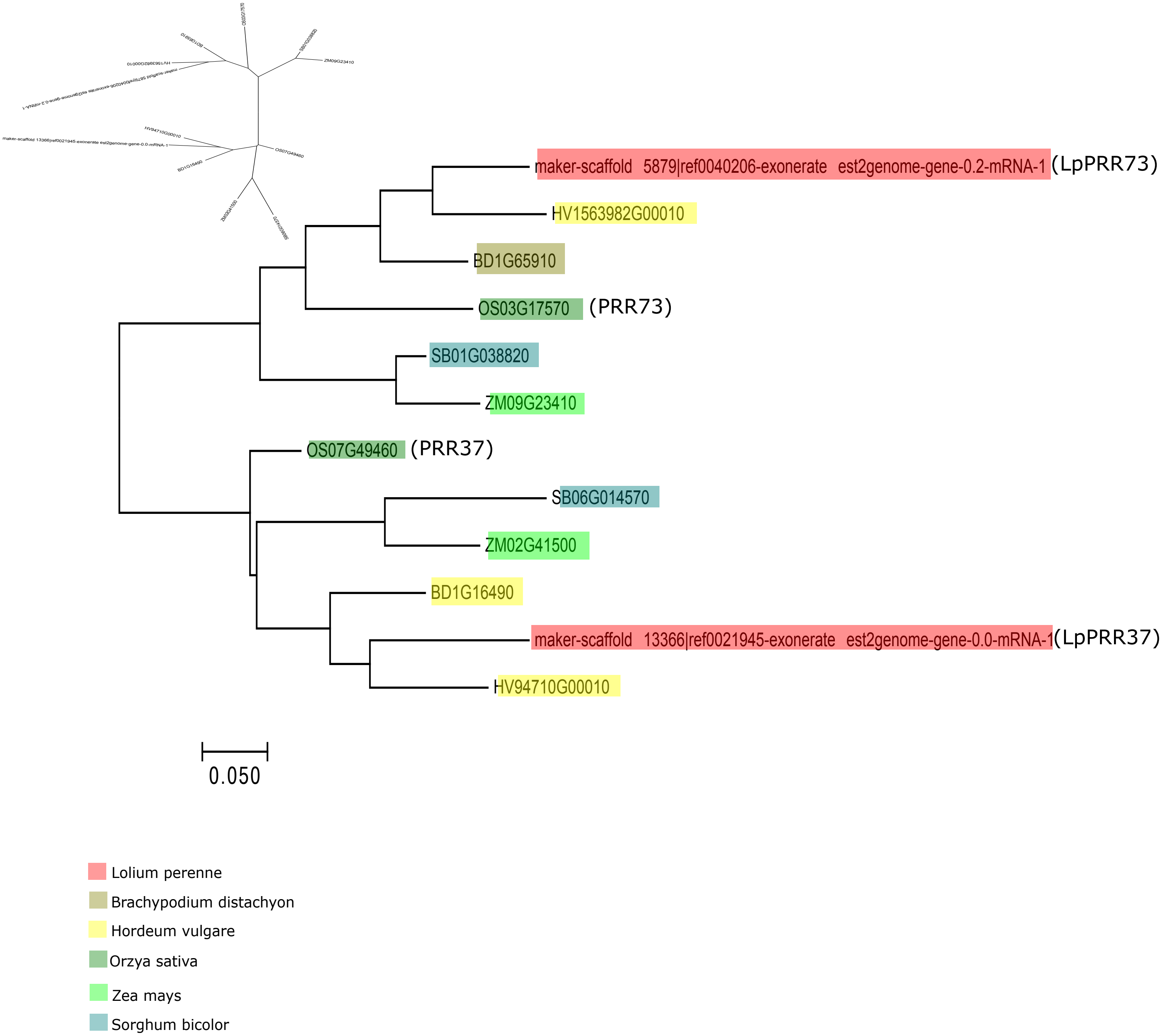

maker-scaffold 5879|ref0040206-exonerate est2genome-gene-0.2-mRNA-1 (LpPRR73)
HV1563982G00010
BD1G65910
OS03G17570 (PRR73)
SB01G038820
ZM09G23410
OS07G49460 (PRR37)
SB06G014570
ZM02G41500
BD1G16490
maker-scaffold 13366|ref0021945-exonerate est2genome-gene-0.0-mRNA-1 (LpPRR37)
HV94710G00010
0.050
Lolium perenne
Brachypodium distachyon
Hordeum vulgare
Orzya sativa
Zea mays
Sorghum bicolor
