# Supplementary material for: Markers associated with heading and aftermath heading in perennial ryegrass full-sib families
Source: BMC Plant Biol. 2016 Jul 16;16:160. doi: 10.1186/s12870-016-0844-y (PMC4947259; doi:10.1186/s12870-016-0844-y)
Supplement: Additional file 11 — Figure S11. Phylogenetic tree of candidate heading gene GI. The evolutionary history was inferred by using the Maximum Likelihood method based on the JTT matrix-based model [67]. The tree is mid-point rooted, drawn to scale, with branch lengths proportional to the number of substitutions per site. All positions containing gaps and missing data were eliminated. Evolutionary analyses were conducted in MEGA 6.06 [66]. Associated Lolium and Arabidopsis proteins were highlighted. (PDF 37 kb) [file 12870_2016_844_MOESM11_ESM.pdf]

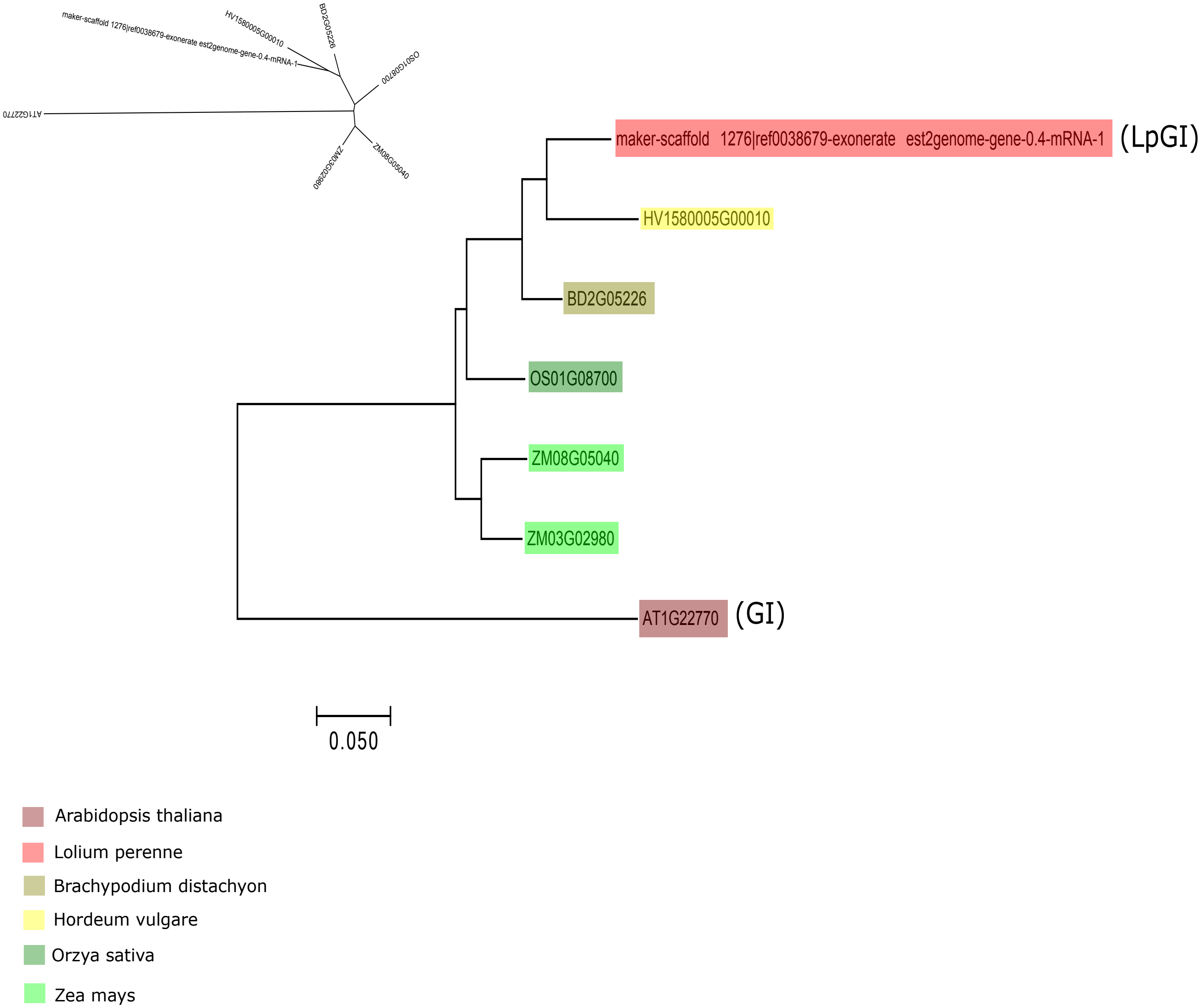
maker-scaffold 1276|ref0038679-exonerate est2genome-gene-0.4-mRNA-1
HV1580005G00010
BD2G05226
OS01G08700
AT1G22770
ZM03G02980
ZM08G05040
maker-scaffold 1276|ref0038679-exonerate est2genome-gene-0.4-mRNA-1
(LpGI)
HV1580005G00010
BD2G05226
OS01G08700
ZM08G05040
ZM03G02980
AT1G22770
(GI)
0.050
Arabidopsis thaliana
Lolium perenne
Brachypodium distachyon
Hordeum vulgare
Orzya sativa
Zea mays
